# High Room-Temperature Magnesium Ion Conductivity in Spinel-Type MgYb_2_Se_4_ Solid Electrolyte

**DOI:** 10.1021/acs.chemmater.5c00131

**Published:** 2025-05-03

**Authors:** Clarissa Glaser, Mohsen Sotoudeh, Manuel Dillenz, Kanchan Sarkar, Jasmin S. Bark, Shashwat Singh, Zhixuan Wei, Sylvio Indris, Riccarda Müller, Kerstin Leopold, Linda F. Nazar, Axel Groß, Jürgen Janek

**Affiliations:** † Institute of Physical Chemistry and Center for Materials Research (ZfM), Justus Liebig University Giessen, Heinrich-Buff-Ring 17, Giessen 35392, Germany; ‡ Institute of Theoretical Chemistry, Ulm University, Albert-Einstein-Allee 11, Ulm 89081, Germany; § Department of Chemistry and the Waterloo Institute for Nanotechnology, University of Waterloo, Waterloo N2L 3G1, Ontario, Canada; ∥ Institute for Applied Materials-Energy Storage Systems (IAM-ESS), Karlsruhe Institute of Technology (KIT), Hermann-von-Helmholtz-Platz 1, Eggenstein-Leopoldshafen 76344, Germany; ⊥ Institute of Analytical and Bioanalytical Chemistry, Ulm University, Ulm 89081, Germany

## Abstract

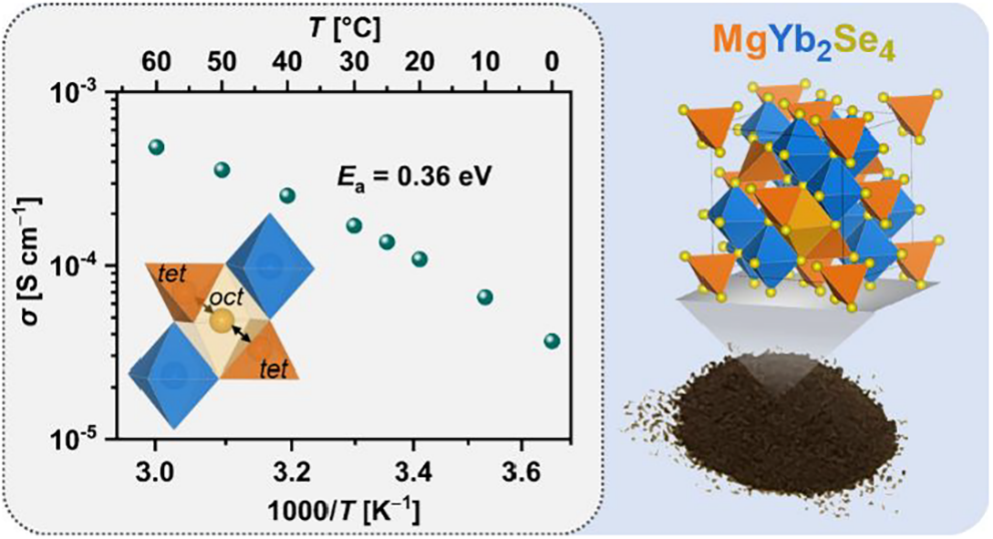

We present three magnesium selenide spinels, MgSc_0.4_Y_0.4_Er_0.4_Tm_0.4_Yb_0.4_Se_4_, Mg_0.75_Sc_2_Se_3.5_Br_0.5_ and MgYb_2_Se_4_, as potential solid electrolytes (SE) for magnesium batteries. In particular, the latter spinel exhibits a room-temperature ionic conductivity exceeding 10^–4^ S cm^–1^ and a low Mg^2+^ migration barrier of 364 meV. The high ionic mobility is attributed to the low magnesium insertion energy revealed by density functional theory (DFT), which indicates a weak binding interaction between magnesium ions and the host lattice. Furthermore, like the two multicomponent spinels, MgYb_2_Se_4_ exhibits lower electronic conductivity compared to previously studied MgB_2_Se_4_ spinels (B = Sc, Y, Er, Tm) and a good electrochemical stability, making it a strong candidate for Mg^2+^ SE applications.

## 1 Introduction

Rechargeable magnesium batteries (RMBs) are considered as promising candidates for next-generation energy-storage solutions since they are expected to be more sustainable than today’s widely used lithium-ion batteries. This is based on the high abundance of magnesium in the earth’s crust (10^4^ times more often than lithium) and its twice to five-times higher volumetric capacity of 3833 mAh cm^–3^ compared to Li metal (2066 mAh cm^–3^) and LiC_6_ (760 mAh cm^–3^) negative electrodes, respectively, caused by the higher charge density of the Mg-ion.^1^ In addition, magnesium has a low standard reduction potential (−2.37 V vs SHE) – and in contrast to Li – a low tendency for dendrite formation during plating.^1,2^

However, the development of room-temperature SEs for solid-state Mg batteries is challenging, as Mg^2+^ ions have a sluggish mobility in the solid state due to their high charge density.^3^ Thus, most of the reported Mg SEs, such as oxides (NASICON-type Mg_0.5_Zr_2_(PO_4_)_3_ and modifications),^4−7^ sulfides (MgS-P_2_S_5_–MgI_2_ solid solutions)^8^ and halides (MgAl_2_Cl_8–*y*_Br_*y*_),^9^ do not reach conductivities higher than 3 × 10^–5^ S cm^–1^ at ambient conditions.^10,11^ The only reported Mg^2+^ SEs that have achieved a room-temperature Mg-ion conductivity σ_ion_ > 10^–4^ S cm^–1^ are the magnesium borohydride derivatives *β*-Mg(BH_4_)·CH_3_NH_2_ (σ_ion_ = 1.5 × 10^–4^ S cm^–1^) and Mg(BH_4_)·1.5NH_3_-60 wt %TiO_2_ (σ_ion_ = 3.0 × 10^–4^ S cm^–1^).^12,13^ Nevertheless, these compounds show a relatively low electrochemical stability (≤1.3 V vs Mg^2+^/Mg), making it challenging for the pairing with suitable cathodes.

Instead, the recently studied MgB_2_Se_4_ (B = Sc, Y, Er, Tm) spinels show oxidation stabilities >3.7 V – sufficient for most transition metal chalcogenide cathodes – and roughly comparable ionic conductivities (σ_ion_ = 2 to 7 × 10^–5^ S cm^–1^ at 25 °C).^14^ This class of SEs benefits from the spinel-type structure, which provides three-dimensional (3D) conduction pathways and a relatively large distance between the migrating Mg^2+^ in its transition state and the neighboring B-cations.^15^ Along with this, the increased polarizability of the Se anions compared to their oxygen and sulfur counterparts weakens the bonding interactions with the Mg-ions, resulting in higher conductivities and lower Mg^2+^ migration barriers *E*_a_.^16,17^ The *E*_a_ values are predicted to be between 290 and 375 meV for several d^0^-metal- and lanthanide-based MgB_2_Se_4_ (B = Sc, Lu, Tm, Er, Y, Ho, Dy, Tb, Sm, Pm, Nd, Pr, La) spinels calculated by density functional theory (DFT).^18,19^ However, both computational and synthesis results suggest that only compounds with an ionic radius *r*_B_ of the B metal between those of Sc and Ho (*r*_Sc–Ho_ = 0.861–0.901 Å) favor the spinel structure in the ground state and possess sufficient stability.^18,20−22^ Interestingly, the compound MgYb_2_Se_4_ (*r*_Yb_ = 0.868 Å) is included in this series of potentially stable spinels,^20,22^ but has never been considered as Mg-ion SE in any computational or electrochemical study.

Also, multicomponent magnesium spinels with multielement substitution on a single crystallographic site (B-site or Se-site) have not yet been investigated. Multicomponent solid electrolytes with increased compositional or lattice site disorder over their ordered, single-component counterparts have recently demonstrated improved ion transport properties.^23−28^ For instance, multicationic and multianionic lithium argyrodites such as Li_6.5_[P_0.25_Si_0.25_Ge_0.25_Sb_0.25_]S_5_I,^23^ Li_6.6_P_0.4_Ge_0.6_S_5_I^24^ and Li_6_PS_5_[Cl_0.33_Br_0.33_I_0.33_]^25^ showed lower activation energies (*E*_a_ = 0.19–0.29 eV) and higher room-temperature ionic conductivities (σ_ion_ = 10^–3^–10^–2^ S cm^–1^) compared to the anion-ordered Li_6_PS_5_I structure (*E*_a_ = 0.38 eV, σ_ion_ = ∼10^–6^ S cm^–1^).^24^ This success makes it promising to apply multielement substitution also to stable MgB_2_Se_4_ spinels in order to improve Mg transport properties, possibly realized with compounds such as MgSc_0.4_Y_0.4_Er_0.4_Tm_0.4_Yb_0.4_Se_4_ and Mg_1–0.5*x*_Sc_2_Se_4–*x*_Br_*x*_ (0 < *x* ≤ 1).

For these reasons, in the present work we expand the family of experimentally investigated MgB_2_Se_4_ (B = Sc, Y, Er, Tm) spinels with MgYb_2_Se_4_ and the above-mentioned multicationic/multianionic compounds (meanly discussed in the Supporting Information) as potential Mg-ion SEs. Among them, MgYb_2_Se_4_ was found to exhibit the highest ionic conductivity and the lowest Mg-ion migration barrier, and also a lower electronic conductivity compared with the prior studied mixed-conducting spinels. The resulting lower electronic transference number as well as the evidence of high electrochemical stability make this spinel an attractive candidate for Mg-ion SEs. We hope that this work, along with its theoretical considerations, motivates further advancement in the development of room-temperature Mg-ion conductors for Mg batteries.

## 2 Results and Discussion

### 2.1 Structural Information

**Figure 1:**
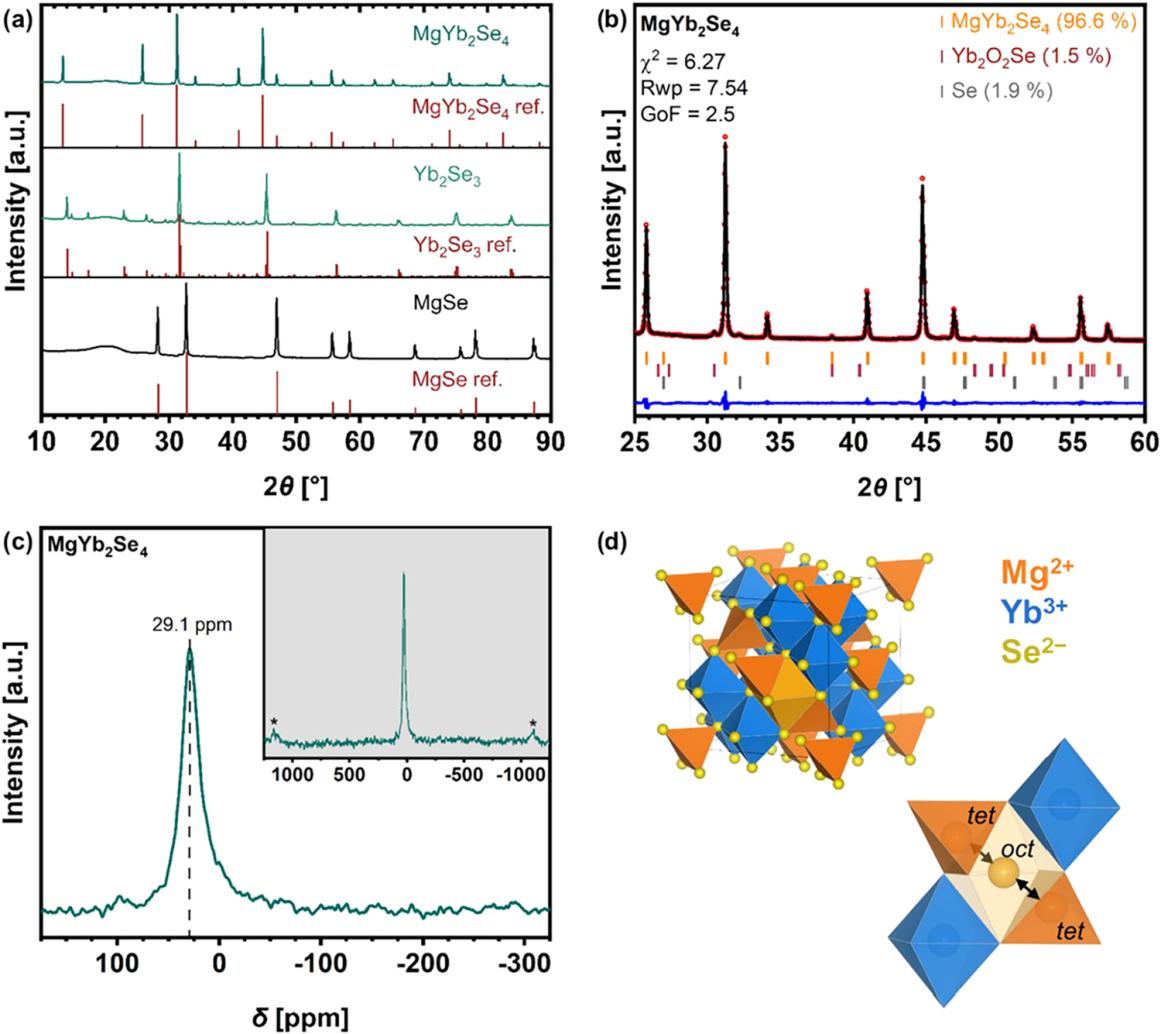
(a) XRD patterns of synthesized MgSe, Yb_2_Se_3_ and MgYb_2_Se_4_, and (b) Rietveld refinement against the XRD data for MgYb_2_Se_4_. Observed and calculated patterns are shown in red and black, and the difference curve is shown in blue. (c) The ^25^ Mg solid-state NMR spectrum, collected at a magnetic field of 11.7 T and a spinning speed of 35 kHz, confirms the Mg position on the tetrahedral site in MgYb_2_Se_4_. The spinning sidebands are marked with an asterisk. (d) Crystal structure of MgYb_2_Se_4_ spinel and the *tet-oct-tet* migration pathway for Mg^2+^. Panel (d) adapted with permission from ref [^14^]. Copyright 2024, Glaser et al.^14^

The MgYb_2_Se_4_ spinel was synthesized under vacuum according to our reported two-step solid-state synthesis.^14^ In the first step, the precursors MgSe and Yb_2_Se_3_ were prepared by reacting stoichiometric amounts of the corresponding elements at 750 and 800 °C, respectively. In the second step, the spinel was formed by a 1:1 reaction of the precursors at 950 °C. As a result, Figure 1a shows that the X-ray diffraction (XRD) pattern of the synthesized spinel and precursors are in excellent agreement with the corresponding Bragg reflections of the reference structures. In addition, Rietveld refinement based on the XRD data of the MgYb_2_Se_4_ sample (Figure 1b, crystallographic information in Table S1) confirms that the material adopts a cubic spinel structure with the *Fd*3̅*m* space group and was obtained in a high purity (96.6 wt %). The minor fraction of impurity was identified as unreacted selenium (1.9 wt %) and Yb_2_O_2_Se (1.5 wt %), whose formation must be due to an as yet unknown oxygen source, which could not be observed in the binary precursor materials (cf. Figure S1).

To gain deeper insight into the MgYb_2_Se_4_ structure, we employed ^25^Mg magic-angle spinning nuclear magnetic resonance (MAS NMR) spectroscopy, a technique that characterizes the local environment and coordination of Mg ions within the material. The ^25^MAS NMR spectrum (Figure 1c) of MgYb_2_Se_4_ shows only a single isotropic peak at 29 ppm, indicating a single coordination environment for Mg. Combined with the XRD refinement results, confirming a normal spinel structure, Mg therefore exclusively occupies the tetrahedral site. Consequently, the MgYb_2_Se_4_ spinel structure (illustrated in Figure 1d) has the Mg^2+^-ions on the 8a sites (tetrahedral (*tet*)) and the Yb^3+^-ions on the 16d sites (octahedral (*oct*)). This results in an ideal element stoichiometry of Mg/Yb/Se (1:2:4), which was also corroborated by energy dispersive X-ray spectroscopy (EDS) analysis of the powder with particles ranging from 1 to 100 μm in size (Figure S2).

### 2.2 Electronic Conductivity

**Figure 2:**
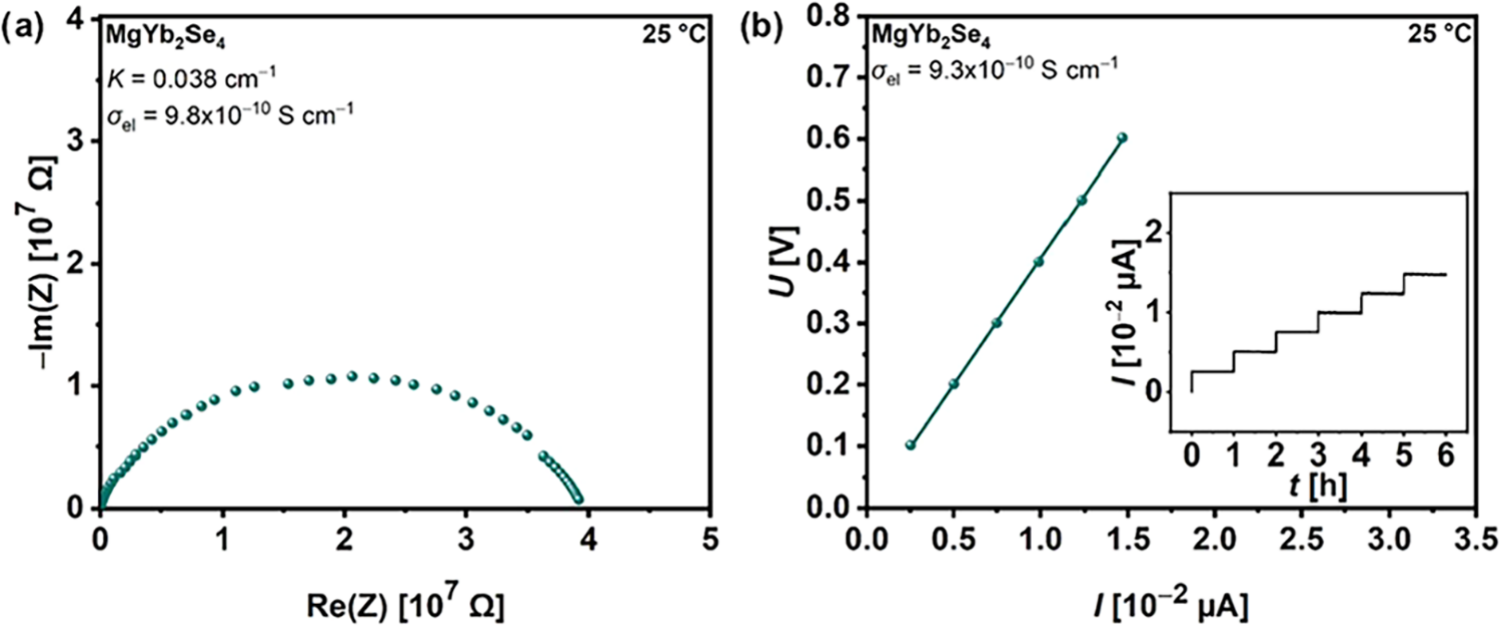
(a) Nyquist plot of the impedance data of a C|MgYb_2_Se_4_|C symmetric ion-blocking cell in the frequency range from 3 MHz to 100 mHz at 25 °C. *K* represents the cell constant, as described in eq 1. (b) DC polarization data at 25 °C obtained for a C|MgYb_2_Se_4_|C cell. During the measurement, different voltages (0.1, 0.2, 0.3, 0.4, 0.5, and 0.6 V) were held for 1 h each. The steady-state current at the end of each holding step (shown in the inset) was plotted against the corresponding voltage to calculate the electronic resistance *R*_el_ of MgYb_2_Se_4_ using a linear fit.

The room-temperature partial electronic conductivity of MgYb_2_Se_4_ was evaluated using electrochemical impedance spectroscopy (EIS) in a C|MgYb_2_Se_4_|C symmetric cell employing ion-blocking carbon (C) electrodes. Consistent with previous studies on MgB_2_Se_4_ spinels,^14,29^ a depressed semicircle without a low-frequency tail appeared in the Nyquist plot (Figure 2a), indicating the presence of an electronic current path in parallel to the ionic one. In principle, this results in two semicircles, representing the parallel combination of the ionic resistance and the geometrical capacitance at high frequencies, and the parallel combination of the electronic resistance and the geometrical capacitance at lower frequencies.^30^ If the ionic conductivity is much larger than the electronic conductivity (σ_ion_ ≫ σ_el_), the low-frequency semicircle is considerable larger than the high-frequency semicircle, whose resistance approaches to zero in the extreme cases. Thus, the observed semicircle is solely attributed to the electronic resistance and geometrical capacitance. The absence of an interface capacitance or a blocking tail at low frequencies confirms this behavior based on the lack of electron blockage.^14^ Consequently, the total resistance of the semicircle corresponds to the electronic resistance, *R*_el_, which is used to calculate the electronic conductivity, σ_el_, by eq 1, applicable to both electronic and ionic conduction
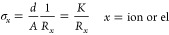
1*d* and *A* represent the thickness and the contact area of the spinel pellet, respectively, and are summarized in the cell constant *K*.

A significant outcome of this study is the determination of the electronic conductivity of MgYb_2_Se_4_, evaluated by impedance measurements as σ_el_ = 9.8 × 10^–10^ S cm^–1^, and further validated by additional DC polarization measurements (Figure 2b). Notably, this value is over an order of magnitude lower than those of the previously studied spinels, including MgSc_2_Se_4_, MgY_2_Se_4_, MgEr_2_Se_4_, and MgTm_2_Se_4_.^14,29^ Therefore, at this point MgYb_2_Se_4_ seems to be a better potential SE, even if the present state of knowledge on the rarely studied MgB_2_Se_4_ spinels does not yet allow the identification of a certain cause for its lower σ_el_ value. According to prior studies on MgSc_2_Se_4_, there are only two hypotheses for the emergence of the electronic conductivity. Canepa et al. proposed in their study that the electronic conductivity arises either from the existence of intrinsic defects neutralized by electrons, or from the presence of electronic conducting secondary phases in the prepared material.^19,31^ The latter hypothesis was also supported by Kundu et al., who further specified that electronic transport possibly occurs via electron jumping/tunneling between well-distributed electron-conducting inclusions (e.g., Sc/ScSe), through the low-conducting spinel matrix.^32,33^ Following these two hypotheses, the electronic conductivity of the MgB_2_Se_4_ spinels is probably dependent on the interplay of various factors, including the extent of secondary phases/defects, the nature of secondary phases/defects (electron-conducting or not) and their distribution in the material. Since these factors partly depend on the specific elemental composition of the spinels and are still partly unknown, this makes it difficult to draw conclusions about the lower electronic conductivity of MgYb_2_Se_4_ in comparison to the other MgB_2_Se_4_ spinels investigated so far.

As with MgYb_2_Se_4_, two of the synthesized multicationic and multianionic spinels, MgSc_0.4_Y_0.4_Er_0.4_Tm_0.4_Yb_0.4_Se_4_ and Mg_0.75_Sc_2_Se_3.5_Br_0.5_, obtained in high phase purity (Figures S3–S5 and S7–S11), also demonstrate low σ_el_ values (σ_el_ = 3.3 × 10^–9^ and 3.6 × 10^–13^ S cm^–1^, Figures S6 and S12). In particular, the latter exhibits an electronic conductivity that is almost five orders of magnitude lower than that of its Br-free, pristine compound MgSc_2_Se_4_ (σ_el_ = 2.0 × 10^–8^ S cm^–1^). This comparison suggests that the substitution of Se with Br counteracts the electronic transport, at least for the specific composition of Mg_0.75_Sc_2_Se_3.5_Br_0.5_. However, the manner in which this is happening can only be speculated at the current stage: On the one hand, this certain degree of aliovalent anion substitution might counteract the formation of the before mentioned intrinsic defects, which possibly induce electronic conductivity.^31^ One the other hand, the aliovalent anion substitution could lead to changes in the electron–phonon interactions of the material.^34^ If the interaction strength exceeds a specific threshold, polarons can form, in which the effective mass of the electrons is increased and thus lower electronic conductivity occurs. Nevertheless, whether either of these two cases is true will need to be investigated in the future.

### 2.3 Ionic Conductivity

The Mg-ion conductivity and the migration barrier along the *tet-oct-tet* migration pathway (Figure 1d) were determined from EIS measurements using symmetric SS|UiO66-MgIL|MgYb_2_Se_4_|UiO66-MgIL|SS cells. These cells contain two charge carrier specific blocking electrodes: ion-blocking stainless-steel (SS) outer electrodes, and electron-blocking interlayers consisting of the pure Mg-ion-conducting ionogel electrolyte UiO66-MgIL [σ_el_(25 °C) = 1.7 × 10^–10^ S cm^–1^,^29^ and σ_ion_(25 °C) = 7.6 × 10^–4^ S cm^–1^, Tables S2–S3]. The latter, the ionogel electrolyte, was prepared from a 1:1.25 (mass ratio) mixture of the metal–organic framework Zr_6_O_4_(OH)_4_(BDC)_6_ with BDC = 1,4-dicarboxylate (denoted as UiO66) and a 1 M Mg(TFSI)_2_ ionic liquid based on [EMIM][TFSI] (denoted as MgIL) and appears as a dry and free-flowing powder since strong adhesive forces holding the viscous MgIL in the UiO66 framework.^29,35^ By using UiO66-MgIL as an interlayer, this ensures better and sufficient physical contact between the rigid, hardly deformable electrodes (here: SS, but also Mg foils) and the rough spinel pellets, and also effectively suppresses the electron transport within the cell, so that the ionic conductivity can be accurately determined from the resulting impedance spectra.^29^

**Figure 3:**
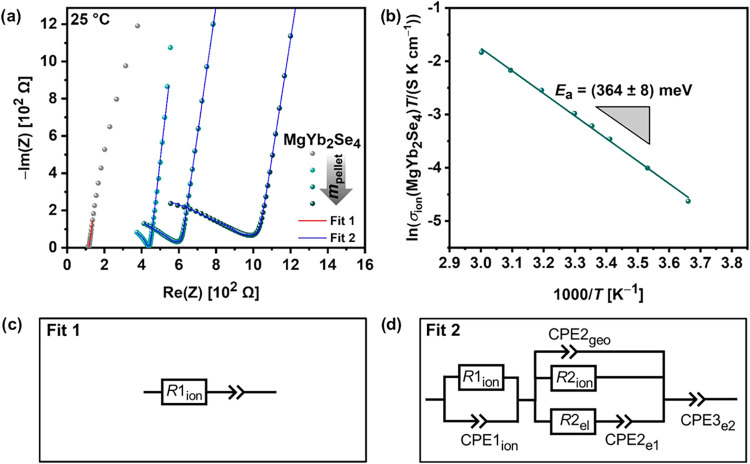
(a) Nyquist plots of SS|UiO66-MgIL|SS reference cell (gray) and SS|UiO66-MgIL|MgYb_2_Se_4_|UiO66-MgIL|SS cells with varied spinel pellet mass/thickness (160 mg/0.46 mm, 220 mg/0.54 mm and 280 mg/0.76 mm) at 25 °C fitted with the equivalent circuits Fit 1 and Fit 2; (b) Arrhenius plot of the average conductivities shown for temperatures from 0 to 60 °C; and (c–d) equivalent circuits Fit 1 and Fit 2 applied to fit the Nyquist plots.

Figure 3a shows the Nyquist plots of room-temperature EIS measurements of cells with varied spinel pellet thicknesses (Table S2) and a reference SS|UiO66-MgIL|SS cell without a spinel pellet. As previously reported,^14,29^ the ionic resistance *R*1_ion_ of the UiO66-MgIL in the reference cell can be obtained by fitting the Nyquist plot with a simple equivalent circuit shown in Figure 3c. The *R*1_ion_ was then adjusted to the UiO66-MgIL interlayer thickness used in the spinel-containing cells (Tables S2–S3 and eq S1), and set as a fixed parameter to determine the ionic resistance *R*2_ion_ of MgYb_2_Se_4_ by applying the equivalent circuit in Figure 3d, which models the impedance of a mixed conductor between charge carrier specific blocking electrodes. By applying eq 1 with *R*2_ion_, an average room-temperature Mg-ion conductivity of σ_ion_ = (1.4 ± 0.3) × 10^–4^ S cm^–1^ is evaluated. This value for MgYb_2_Se_4_ is considerably higher than that of the already investigated MgB_2_Se_4_ (B = Sc, Y, Er and Tm) spinels of the same material class (σ_ion_ = 2 × 10^–5^–7 × 10^–5^ S cm^–1^)^14,29^ and comparable to that of *β*-Mg(BH_4_)·CH_3_NH_2_ (σ_ion_ = 1.5 × 10^–4^ S cm^–1^)^12^ and Mg(BH_4_)·1.5NH_3_-60 wt %TiO_2_ (σ_ion_ = 3.0 × 10^–4^ S cm^–1^).^13^ To our knowledge, these borohydride compounds exhibit the highest room-temperature ionic conductivity among the Mg-ion SEs, even though the latter is not competitive due to its high electronic conductivity (σ_el_/σ_ion_ = 0.6).^13^

Figure 3b shows the Arrhenius plot of the average ionic conductivities of MgYb_2_Se_4_, determined from EIS measurements in the temperature range between 0 and 60 °C (Figures S13–S14). Based on that, the activation energy *E*_a_ = 364 meV was calculated using the Arrhenius equation (eq 2)
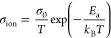
2where σ_0_ is the conductivity pre-exponential factor. This *E*_a_ value is lower than those reported for the other experimentally studied magnesium spinels (381–406 meV)^14,29^ An explanation for the lower *E*_a_ and the higher ionic conductivity of MgYb_2_Se_4_ compared to the other investigated spinels cannot be derived from the experimental data and is not immediately obvious. Thus, we will consider these results in the next section (Section 2.4) from a theoretical point of view.

**Figure 4:**
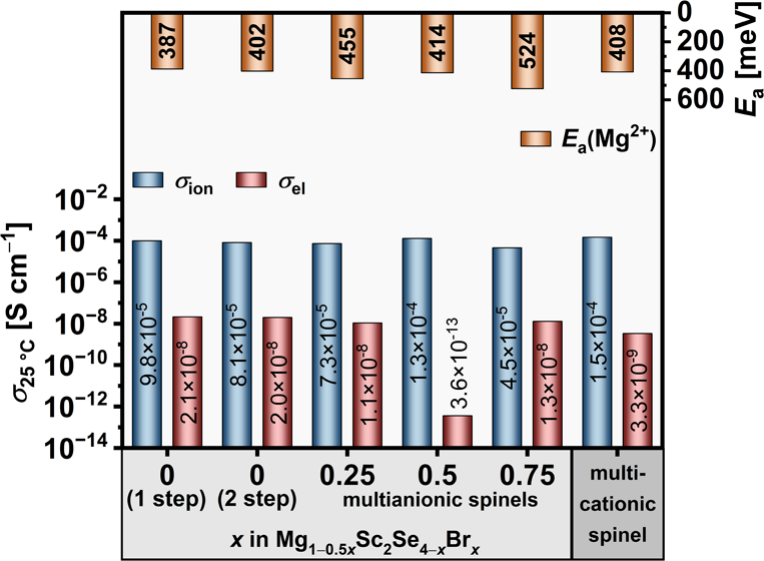
Overview of experimentally determined Mg-ion migration barriers *E*_a_, ionic conductivities σ_ion_ and electronic conductivities σ_el_ of the prepared multicationic and multianionic compounds as well as of one- and two-step synthesized MgSc_2_Se_4_. Data for one-step synthesized MgSc_2_Se_4_ were used from ref (29).

An overview of the transport properties of the multicationic MgSc_0.4_Y_0.4_Er_0.4_Tm_0.4_Yb_0.4_Se_4_ spinel and multianionic Mg_1–0.5*x*_Sc_2_Se_4–*x*_Br_*x*_ (*x* = 0.25, 0.5, 0.75) compounds provides Figure 4. In comparison to the pristine MgSc_2_Se_4_, these SE compounds do not show any significant improvement in their migration barrier *E*_a_ and ionic conductivity σ_ion_, as determined by EIS measurements of SS|MgIL|SE|MgIL|SS cells (Figures S15–S18 and Tables S4–S5). More specifically, the σ_ion_ values remain constant and comparable to that of the pristine MgSc_2_Se_4_ and even decrease for the multianionic compounds with *x* ≥ 0.75, where partial decomposition of the spinel phase occurred (cf. Figure S7). Note that these σ_ion_ values are slightly overestimated because instead of UiO66-MgIL interlayers, glass fiber-MgIL interlayers were used, which wet the surface of the spinel pellets, resulting in higher conductivities. However, since σ_ion_ of MgSc_2_Se_4_ was determined with both, glass fiber-MgIL interlayer (σ_ion_ = 9.8 × 10^–5^ S cm^–1^, this work) and UiO66-MgIL interlayer (σ_ion_ = 2.4–5.5 × 10^–5^ S cm^–1^),^29^ the true σ_ion_ values of the multicationic and multianionic (*x* < 0.75) spinels with similar ionic conductivities can also be assigned to the latter range. This demonstrates that the conducted multielement substitution in MgB_2_Se_4_ spinels does not lead to the desired result of improved ion transport properties.

### 2.4 Computational Investigation

**Figure 5:**
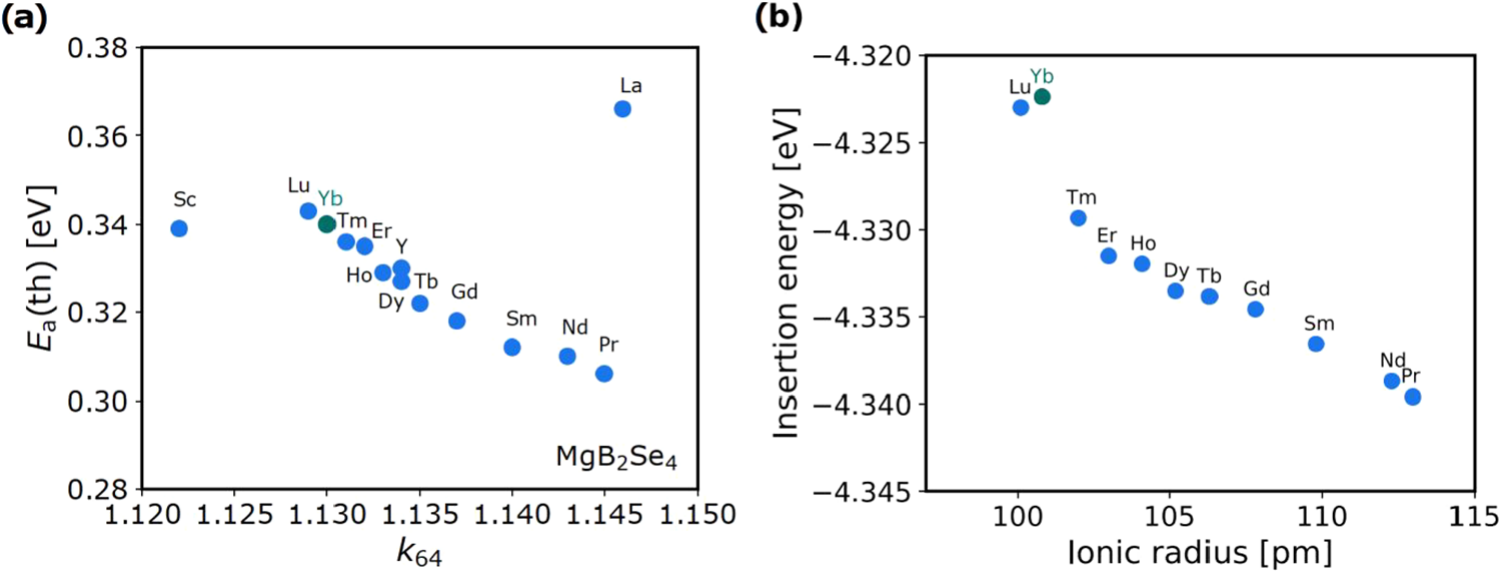
(a) Computationally predicted *E*_a_(th) obtained using a PBE-DFT approach alongside with the NEB method. (b) Insertion energy, relative to a metallic magnesium anode, plotted as a function of ionic radius for MgB_2_Se_4_ compounds. Each compound is represented by a sphere, with the associated transition metal indicated above.

The variation in the theoretically predicted migration barrier *E*_a_(th) of MgB_2_Se_4_ spinels is related to the cationic radii, as the incorporation of larger lanthanides into the spinel structure increases the unit cell volume.^18,36^ In particular, a 17% increase in the radius of the cations from 1.001 Å (Lu^3+^) to 1.172 Å (La^3+^) is associated with an approximate 17% expansion in the unit cell volume. A recent study introduced the distance ratio *k*_64_, which is a bond length relationship between the migrating cation and selenium anions at octahedral (*d*(cn6)) and tetrahedral (*d*(cn4)) coordination sites, as a parameter to quantify the impact of trigonal distortion.^37^ The *k*_64_ value has been demonstrated to be an optimal geometrical descriptor for both the static and kinetic components of *E*_a_(th).^14^Figure 5a illustrates the theoretical migration energy barriers *E*_a_(th) for Mg^2+^ ions in MgB_2_Se_4_ spinels as a function of *k*_64_, which were obtained by DFT calculations alongside with the NEB method, assuming a vacancy diffusion mechanism in the dilute vacancy limit (one Mg vacancy per supercell). For all compounds considered, the calculated *E*_a_(th) value is in the range of 306–366 meV. The larger ratio *k*_64_, which is driven by the inclusion of larger cations, correlates with enhanced Mg^2+^ mobility, as evidenced by a reduction in calculated migration barriers from 343 meV in MgLu_2_Se_4_ to 306 meV in MgPr_2_Se_4_. The reduction in the Mg^2+^ migration barrier can be observed to follow an approximately linear trend with increasing cation radii, with the exception of MgLa_2_Se_4_, where a deviation can be seen to occur due to the destabilization of Mg^2+^ in the octahedral coordination.

These generally low *E*_a_(th) values within the considered range of MgB_2_Se_4_ spinels indicate a high mobility for the Mg-ion in these compounds, but the dependence of the migration barrier on the individual contributing factors is complicated. Our DFT calculations show a linear relationship between the static (site preference energy Δ*E*) and kinetic (kinetically resolved activation energy *E*_KRA_)^38^ components of the migration barrier and the *k*_64_ parameter as shown in Figure S19. To overcome the difficulties associated with the directional dependence of *E*_a_(th), we introduce a kinetically resolved activation energy (*E*_KRA_), which involves calculating the average energy of the initial and final states of the hop and subtracting it from the activation energy. However, the insertion energy of magnesium within the spinel structure, relative to a metallic magnesium anode (eq 3), exhibits distinct behavior across the lanthanides, with ytterbium (Yb) demonstrating the smallest insertion energy, as illustrated in Figure 5b. The insertion energy, *E*_insert_, is defined as
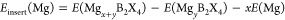
3where *E*(Mg_*y*_B_2_X_4_) represents the total energy of the spinel with a magnesium concentration *y* in the unit cell, and *E*(Mg) is the cohesive energy of bulk magnesium in its metallic phase. In the present study, the variable *y* is determined by the magnesium content of the supercell. This is achieved by removing one Mg atom from a total of eight, thus yielding *y* = 0.875, while ensuring that *x* + *y* = 1 is maintained to preserve charge balance and spinel stoichiometry. The smallest insertion energy observed for Yb suggests that the interactions between magnesium ions and the host lattice are relatively weak in the MgYb_2_Se_4_ crystal structure. Consequently, the weaker binding corresponds to lower probability of “residence” of ions at specific sites within the lattice, thereby facilitating more efficient and rapid ionic transport through the material.^39^

A reduction in the activation barrier from 346 to 306 meV is equivalent to a change in *E*_a_(th) of only 40 meV. At typical operating temperatures (e.g., 300 K), this modest change has a small impact on ionic conductivity, with a conductivity of about 1.7 × 10^–6^ for *E*_a_(th) = 346 meV and 7.7 × 10^–6^ for *E*_a_(th) = 306 meV considering the Boltzmann factor exp(−*E*_a_/*k*_B_*T*), which does not correspond to a full order of magnitude change in conductivity. Note that the calculated conductivity values are dependent on the assumed pre-exponential factor σ_0_, and that variations in the pre-exponential term could lead to significant changes in the conductivity at 300 K, potentially influencing the observed effect of the 40 meV activation energy difference. σ_0_ is associated with a number of factors, including the concentration of charge carriers, the attempt frequency of ionic jumps and the entropy of activation, i.e., the change in the local vibrations around the jumping ion. A reduction in binding energy facilitates a higher ion mobility by lowering the energy required to displace an ion from its equilibrium position, thereby increasing the attempt frequency and overall ionic conductivity. It can therefore be concluded that while alterations in the activation energy predominantly impact the exponential term, reductions in binding energy can result in a less rigid bond, increasing the attempt frequency.

Note that ytterbium also exists in the +2 oxidation state (Yb^2+^), which is characterized by a filled 4f orbital configuration ([Xe] 4f^14^) with additional electronic stability, affecting its ionic radius and interactions with the lattice. We hypothesize that in the context of MgYb_2_Se_4_, the reduction of Yb^3+^ to Yb^2+^ may occur under certain conditions due to the chemical environment and the ability of magnesium to reduce the Yb^3+^ ions, as supported by previous reports of binary compounds such as YbSe forming in similar systems.^20^ For this reason, the calculated low insertion energy in this spinel could be the result of a combination of factors such as the unique electronic structure of Yb, the possible formation of Yb^2+^ ions in the solid state and the associated defect chemistry. However, computational models may inadequately capture the interplay between Yb^2+^ and compensating defects or vacancies in the crystal structure. This can lead to an overestimation of the energy barrier for ion migration. These effects are different from those observed for Tm, Er, Y, and Sc which typically adopt the +3 oxidation state and have an unfilled shell, resulting in different bonding and migration properties.

### 2.5 Cycling Performance and Electrochemical Stability

**Figure 6:**
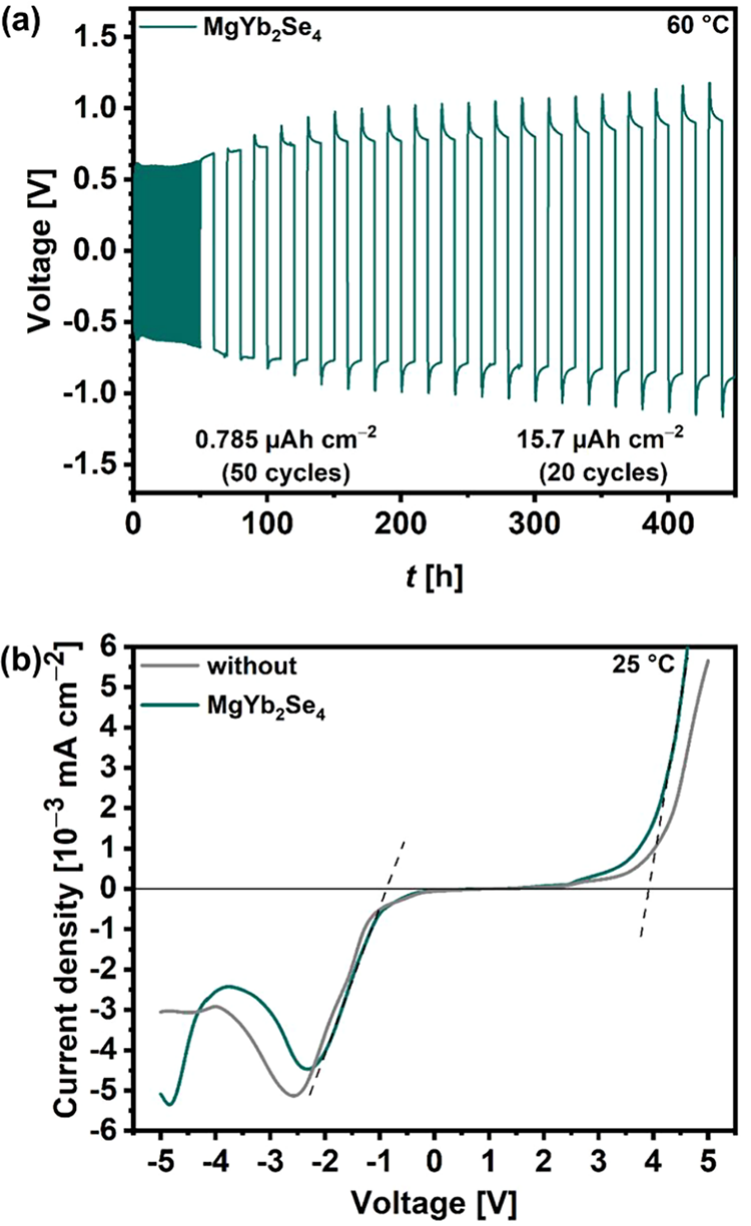
(a) Long-term cycling performance during Mg plating/stripping of a Mg|UiO66-MgIL|MgYb_2_Se_4_|UiO66-MgIL|Mg cell at 60 °C by applying a current density of ±1.57 μA cm^–2^, and (b) LSV curves of Mg|UiO-66-MgIL|SS cells (“without”) and Mg|UiO-66-MgIL|MgYb_2_Se_4_|UiO66-MgIL|SS cells recorded at a scan rate of −0.1/0.1 mV s^–1^ at room-temperature.

To further investigate the MgYb_2_Se_4_ spinel as potential Mg-ion SE, the cycling performance and electrochemical stability of the sandwich-type UiO66-MgIL|MgYb_2_Se_4_|UiO66-MgIL pellets against Mg metal were evaluated. The Mg plating-stripping cycling was performed with a symmetric Mg|UiO66-MgIL|MgYb_2_Se_4_|UiO66-MgIL|Mg cell at 60 °C. As shown in Figure 6a, the cell was initially activated over 50 cycles at a current density of 1.57 μA cm^–2^ and a dwell time of 30 min for each plating or stripping step (plated charge amount of 0.785 μAh cm^–2^ ≙ ideally 2 nm of Mg). Afterward, the plated charge was increased by a factor of 20 (≙ 41 nm of Mg for each step of 10 h) for further 20 cycles. Reversible plating-stripping cycling at an increasing overpotential from 0.6 to 1.2 V was observed with this amount of charge. Since the overpotential increases and exceeds the estimated *IR* drop of 0.21 mV across the sandwiched pellet (spinel: *IR* = 0.18 mV, *d* = 54 mm, σ_ion_ = 4.8 × 10^–4^ S cm^–1^ and UiO66-MgIL: *IR* = 0.03 mV, *d* = 52 mm, σ_ion_ = 2.5 × 10^–3^ S cm^–1^) this clearly indicates interface-dominating resistances inside the cell.

Linear sweep voltammetry (LSV) was carried out with asymmetric two-electrode cells, where one Mg foil served as both the counter and reference electrode, and a stainless-steel current collector acted as the working electrode. The oxidation/reduction onset potential was measured with a scan rate of −0.1 or 0.1 mV s^–1^ from open circuit voltage (OCV) to −5 or 5 V (vs Mg^2+^/Mg). As shown in Figure 6b, the onset potentials for reduction and oxidation currents were observed at −0.9 and 3.9 V, respectively, consistent with those of the earlier reported spinels (Figure S20),^14^ but also similar to the spinel-free reference cell. This indicates that the spinel’s stability possibly exceeds these specified limits, as these are constrained by the decomposition of the UiO66-MgIL electrolyte. To determine the presumably larger range of the electrochemical stability window, it is therefore necessary to cycle the cells without UiO66-MgIL interlayers. Unfortunately, due to the poor physical contact between the stiff SS/Mg electrodes and the spinel pellet,^29^ this is not feasible at the present until suitable electrodes have been developed that can adapt well to the solid electrolyte surface. Nevertheless, these first results already demonstrate that the preliminary oxidation stability of 3.9 V is in any case higher than that of the borohydride SEs (≤1.3 V vs Mg^2+^/Mg^2^),^12,13^ which considerably broadens the selection of cathode materials for the spinels.

## 3 Conclusions

In summary, we present three spinel-type phases, MgSc_0.4_Y_0.4_Er_0.4_Tm_0.4_Yb_0.4_Se_4_, Mg_0.75_Sc_2_Se_3.5_Br_0.5_ and MgYb_2_Se_4_, with lower electronic conductivity (σ_el_ = 3.3 × 10^–9^, 3.6 × 10^–13^, and 9.8 × 10^–10^ S cm^–1^) than previously reported for magnesium selenide spinels (σ_el_ > 10^–8^ S cm^–1^). Among them, MgYb_2_Se_4_ exhibits a relatively low Mg^2+^ migration barrier (364 meV) and a very high room-temperature ionic conductivity (σ_ion_ = 1.4 × 10^–4^ S cm^–1^). The theoretical calculations offer no simple and straightforward explanation for the low activation barrier and relatively high ionic conductivity. The tendency of ytterbium to also form Yb^2+^ ions in the solid state and corresponding defects, though not proven in our case, may be one factor. According to our calculations, MgYb_2_Se_4_ shows an anomaly in the magnesium insertion energy, i.e., easier reduction by magnesium, that might be related to the tendency of Yb^3+^ ions to be reduced to Yb^2+^. However, how this affects the magnesium mobility needs to considered with more advanced theoretical calculations. Future studies using first-principles molecular dynamics and defect thermodynamics calculations may provide a more complete understanding of the potential role of Yb oxidation states and defect interactions in facilitating magnesium ion transport in MgYb_2_Se_4_. Finally, the resulting low electronic transference number (7 × 10^–6^) of MgYb_2_Se_4_ in contrast to MgSc_2_Se_4_, MgY_2_Se_4_, MgEr_2_Se_4_, and MgTm_2_Se_4_ (5 × 10^–3^ – 0.2) coupled with its high electrochemical stability window (−0.9 to 3.9 V vs Mg^2+^/Mg) make this spinel a highly attractive candidate as a Mg-ion SE.

## 4 Experimental Section

### 4.1 Materials

magnesium powder (Sigma-Aldrich, ≥99%), ytterbium chips (chemPUR, 99.9% REO), selenium powder (Alfa Aesar, 99.999%), scandium bromide (ScBr_3_, Fisher Scientific, ultra dry, 99.99%), scandium selenide (Sc_2_Se_3_, as-prepared^29^), thulium selenide (Tm_2_Se_3_, as-prepared^14^), erbium selenide (Er_2_Se_3_, as-prepared^14^) and yttrium selenide (Y_2_Se_3_, as-prepared^14^), magnesium scandium selenide (MgSc_2_Se_4_, as-prepared^29^), magnesium bis (trifluoromethanesulfonyl) imide (Mg(TFSI)_2_, TCI, >97%), 1-ethyl-3-methylimidazolium bis (trifluoromethanesulfonyl) imide ([EMIM][TFSI], TCI, >98%), metal–organic framework (Zr_6_O_4_(OH)_4_(BDC)_6_ with BDC = 1,4-dicarboxylate, denoted as UiO66 and as-prepared^35^), Mg foil (chemPUR, 99.98%), super P (MSE Supplies, >99%), glass fiber separators (Whatmann GF/A).

### 4.2 Synthesis of MgYb_2_Se_4_ Spinel

First, the binary selenides MgSe and Yb_2_Se_3_ were prepared. To synthesize MgSe, a stoichiometric mixture of magnesium and selenium powder were hand-milled for 15 min, and 0.5 g of the powder mixture was pressed into a pellet (Ø = 10 mm) by applying an isostatic pressure of 300 MPa for 30 min. Then, the pellet was vacuum sealed in a quartz glass ampule (first baked out 800 °C for 2 h under dynamic vacuum) and heated at 750 °C for 24 h (180 °C h^–1^ heating rate) in a furnace (Nabertherm with controller P 300). The Yb_2_Se_3_ synthesis was carried out with stoichiometric amounts of selenium powder and ytterbium metal chips. The selenium powder was placed on the bottom of a graphite crucible and covered with the metal chips. After vacuum sealing the crucible in a quartz glass ampule, the reaction was performed at 800 °C for 48 h (60 °C h^–1^ heating rate). In the next step, a stoichiometric mixture of MgSe and Yb_2_Se_3_ was hand-milled and pressed to a 0.5 g pellet using the same conditions as described above. To finally obtain the MgYb_2_Se_4_ spinel, the pellet was vacuum sealed in an ampule and heated at 950 °C for 20 h (180 °C h^–1^ heating rate). All preparations and sample treatments were performed under dry argon atmosphere (glovebox) or in vacuum and all reactants were used as received.

### 4.3 Synthesis of MgSc_0.4_Y_0.4_Er_0.4_Tm_0.4_Yb_0.4_Se_4_ Spinel Solid Solution

A stoichiometric mixture (5:1:1:1:1:1) of MgSe, Sc_2_Se_3_, Y_2_Se_3_, Er_2_Se_3_, Tm_2_Se_3_ and Yb_2_Se_3_ was hand-milled and pressed to a 0.5 g pellet. Then, the pellet was vacuum sealed in an ampule and heated at 950 °C for 30 h (180 °C h^–1^ heating rate).

### 4.4 Synthesis of Mg_1–0.5*x*_Sc_2_Se_4–*x*_Br_*x*_ (*x* = 0.25, 0.5, 0.75, 1)

Mixtures of (1–0.5*x*) MgSe, (1–1/6*x*) Sc_2_Se_3_ and (1/3*x*) ScBr_3_ were hand-milled and pressed to 0.5 g pellets. Afterward, each pellet was vacuum sealed in an ampule and heated at 950 °C for 24 h (180 °C h^–1^ heating rate).

### 4.5 X-ray Diffraction (XRD)

Powder XRD patterns of the samples were collected on an Empyrean powder diffractometer (Malvern PANalytical Ltd.). Each sample was placed on top of a silicon zero background holder and sealed with Kapton polyimide film under argon atmosphere to avoid contact with air and humidity. Using Cu Kα radiation the diffraction data was recorded in the 2*θ* range from 5 to 90° with a step size of 0.026° and a counting time per step of 200 s. References taken from ICSD Inorganic Crystal Structure Database: MgSe (ICSD 53946),^40^ YbSe (ICSD 106048),^41^ Yb_2_Se_3_ (ICSD 652191),^42^ MgYb_2_Se_4_ (ICSD 76053),^43^ Yb_2_O_2_Se (ICSD 25811),^44^ Se (ICSD 164263),^45^ Se (ICSD 23072),^46^ Se (ICSD 40016),^47^ Sc_2_Se_3_ (ICSD 651804),^42^ MgSc_2_Se_4_ (ICSD 642814),^43^ Tm_2_Se_3_ (ICSD 652078),^42^ MgTm_2_Se_4_ (ICSD 76051),^43^ Er_2_Se_3_ (ICSD 79227),^48^ MgEr_2_Se_4_ (ICSD 630754),^43^ Y_2_Se_3_ (ICSD 652183)^49^ and MgY_2_Se_4_ (ICSD 76052).^43^ Reference data for ScBr_3_ (mp-1186984) were retrieved from the Materials Project from database version v2023.11.1.^50^

### 4.6 Rietveld Analysis

Rietveld refinement against the MgYb_2_Se_4_ XRD data was performed using the software FullProf Suite version January 2021. Starting models for the Rietveld refinement were taken from references listed in Section 4.5.

### 4.7 Flame Atomic Absorption Spectrometry (FAAS)

Quantification of Mg was performed using the flame unit of an AA-7000 spectrometer (Shimadzu Corporation, Japan) equipped with a Mg hollow cathode lamp at 285.2 nm and ASC-7000 liquid auto sampler (Shimadzu Corporation, Japan). Synthetic air (15.0 L min^–1^; MTI, Neu-Ulm, Germany) and acetylene (1.8 L min^–1^; MTI, Neu-Ulm, Germany) were used as fuel gases. Quantification was achieved by means of external calibration in the range from 0.2 to 1.0mg L^–1^.

### 4.8 Total Reflection X-ray Fluorescence Spectrometry (TXRF)

For the measurements high-efficiency module S2 Picofox (Bruker Nano GmbH, Berlin, Germany) equipped with Mo X-ray tube was used. The measurement lifetime was set to 1000 s and excitation of the sample was carried out using a voltage of 50 kV and a current of 600 μA. For quantification Cr internal standard solution (1000 mg L^–1^; Merck KGaA, Germany) was used. Spectra PicoFox (7.2.5.0, Bruker Nano GmbH) software was used to evaluate the obtained spectra and for deconvolution the profile Bayes normal fit was selected.

### 4.9 Scanning Electron Microscopy (SEM) and Energy-Dispersive X-ray Spectroscopy (EDS)

SEM images and EDS maps of the samples were recorded with a Gemini SEM 560 high-resolution scanning electron microscope (Carl Zeiss AG, Germany). An acceleration voltage of 15 kV, a working distance of 8.8 mm and an aperture size of 60 μm were used. The SEM images were collected with the in-lens detector and a X-Max50 detector (Oxford Instruments, U.K.) was used for the EDS mapping. To avoid air contamination, the sample transfer was carried out with a Leica EM VCT500 (Leica Microsystems Germany) shuttle.

### 4.10 Nuclear Magnetic Resonance (NMR) Spectroscopy

^25^Mg magic-angle spinning (MAS) NMR spectroscopy was performed at a magnetic field of 11.7 T, corresponding to a resonance frequency of 30.6 MHz. The measurements were carried out in 1.3 mm rotors at a spinning speed of 35 kHz with a rotor-synchronized Hahn-echo pulse sequence and a π/2 pulse duration of 3.7 μs. The recycle delay was 1 s and the spectra were referenced to an aqueous solution of 5 M MgCl_2_.

### 4.11 Cell Assembly and Electrochemical Measurements

All electrochemical measurements were carried out with a VMP300 electrochemical workstation from Bio-Logic Science Instruments SAS and recorded with the corresponding software EC-Lab. The RelaxIS 3 software (RHD Instruments, Darmstadt, Germany) was used to fit the obtained impedance data.

To measure the electronic conductivity σ_el_, a homemade battery cell casing was used, as introduced in an earlier work.^51^ For a typical measurement, 120–160 mg spinel powder was filled in the PEEK housing (Ø = 10 mm) of the cell. Then, a pellet was formed inside the housing by pressing the powder between two stainless-steel stamps (SS) using a hand press. Layers of 24 mg super P (vacuum-dried at 150 °C for 24 h), denoted with C, were inserted between the spinel pellet and the stainless-steel stamps, working as ion-blocking electrodes. For densification, the symmetric C|spinel|C cell was pressed at 3 t for 3 min and subsequently fixed in an aluminum framework with 10 N m torque by means of a screw. The electrochemical impedance spectroscopy (EIS) measurements were carried out in a frequency range from 3 MHz to 100 mHz with a current (AC) amplitude of 10 mV. For the chronoamperometry (CA) measurements the voltage was increased stepwise (0.1, 0.2, 0.3, 0.4, 0.5, and 0.6 V) with 1 h resting time per step. Then, the steady-state current at the end of each step was plotted against the corresponding voltage to calculate the electronic resistance *R*_el_ of the spinel pellet from a linear fit. From the obtained *R*_el_ value of the EIS and CA measurements, σ_el_ was calculated based on eq 1.

To determine the ionic conductivity σ_ion_ and the Mg-ion migration barrier of MgYb_2_Se_4_, different amounts (160, 220, and 280 mg) of spinel powder were pressed in the PEEK housing, analogous to the procedure mentioned above. Purely Mg-ion conducting interlayers made from 40 mg ionogel SE, denoted as UiO66-MgIL, were employed as electron-blocking electrodes. The UiO66-MgIL consisted out of the metal–organic framework (MOF) compound Zr_6_O_4_(OH)_4_(BDC)_6_ with BDC = 1,4-dicarboxylate (denoted as UiO66) impregnated in a mass ratio of 1:1.25 with a 1 M Mg(TFSI)_2_ ionic liquid based on [EMIM][TFSI] (denoted as MgIL), as described in an earlier report.^29^ Then, the symmetrical SS|UiO66-MgIL|MgYb_2_Se_4_|UiO66-MgIL|SS cells were densified as mentioned before. In case of the MgSc_2_Se_4_ and its multicationic/multianionic derivates, 160 mg spinel powder was pressed in the PEEK housing and MOF-free electron-blocking interlayers were employed as less expensive alternative. These interlayers consisted out of glass fiber separators (Ø = 10 mm) moistened with one droplet (5 μL) of MgIL. After fixing all cells in an aluminum framework, EIS measurements were recorded at different temperatures between 0 and 60 °C, with each temperature hold for 1.5 h before the measurement. A frequency range from 3 MHz to 100 mHz and an AC amplitude of 10 mV were chosen. From the obtained resistance from the fitting of the impedance data, the ionic conductivity was calculated according to eq 1.

For the LSV measurements, asymmetric Mg|UiO66-MgIL|MgYb_2_Se_4_|UiO66-MgIL|SS cells were assembled. First, symmetrical cells with 220 mg MgYb_2_Se_4_ powder (or 0 mg for the reference cell) and 40 mg UiO66-MgIL per layer were prepared, analogous to the determination of σ_ion_. Then, a Mg foil (Ø = 9 mm) polished with a scalpel was placed between one UiO66-MgIL layer and the SS stamp. After applying a pressure of 3 t for 1 min to the cell, LSV from open circuit voltage (OCV) to 5 or −5 V, respectively, was carried out with a scan rate of 0.1/–0.1 mV s^–1^ at 25°C and 60 °C.

The Mg plating/stripping measurements were performed with symmetrical Mg|UiO66-MgIL|MgYb_2_Se_4_|UiO66-MgIL|Mg cells. Here, two Mg electrodes were included according to the previous procedure. Then, chronopotentiometric stripping/plating was carried out over 70 cycles (50 cycles with *t*_cycle_ = 1 h and 20 cycles with *t*_cycle_ = 20 h) at 60 °C with a current density of ±0.785 and 1.57μA cm^–2^ per step, respectively.

### 4.12 Computational Details

Periodic density functional theory (DFT)^52,53^ calculations to determine the Mg^2+^ migration barriers in d^0^-metal- and lanthanide-based MgB_2_Se_4_ spinels were performed. We employed the Perdew, Burke, and Ernzerhof (PBE)^54^ exchange and correlation functional and the projector augmented wave method (PAW)^55^ as implemented in the Vienna Ab initio Simulation Package (VASP).^56−58^ The calculations were conducted for the conventional unit cell with 56 atoms and all forces on the atoms converged below 0.01 eV Å^–1^. The atomic composition consists of 8 Mg, 16 Yb, and 32 Se atoms. This enlarged supercell allows a more accurate representation of defects, disorder, and electronic interactions in the material. The total energy was converged below 1 × 10^–5^ eV per supercell, using a 2 × 2 × 2 *k*-point mesh and a cutoff energy of 520 eV.

We employed the Climbing Image Nudged Elastic Band (CI-NEB)^59^ method with four images between tetrahedral and octahedral sites. The complete energy profile was obtained by mirroring at the octahedral site, representing an intermediate site of the migration event. The forces in the NEB calculations were converged to 0.05 eV Å^–1^. All migration barriers were determined in the dilute vacancy limit (one vacancy per supercell), with the volume fixed to the one of the pristine structures.

## Data Availability

The data that support the findings of this study are openly available in Zenodo at https://doi.org/10.5281/zenodo.14610415, reference number 14610415. All electronic structure calculations used in this work are made available under the Creative Commons Attribution license (CC-BY 4.0) on the NOMAD respository (https://nomad-lab.eu) under the following link: https://doi.org/10.17172/NOMAD/2025.05.02-1.

## Supporting Information Available

Additional information on XRD patterns, crystallographic data, SEM images, EDS maps, electrochemical characterization, ionic and electronic conductivities, activation energies, LSV data, Mg plating/stripping data, FAAS data, and TXRF data (PDF)
