# Supplementary material for: High Room-Temperature Magnesium Ion Conductivity in Spinel-Type MgYb2Se4 Solid Electrolyte
Source: Chem Mater. 2025 May 3;37(9):3353–62. doi: 10.1021/acs.chemmater.5c00131 (PMC12080403; doi:10.1021/acs.chemmater.5c00131)
Supplement: Supplementary file 1 — cm5c00131_si_001.pdf [file cm5c00131_si_001.pdf]

High Room-Temperature Magnesium Ion Conductivity in Spinel-Type $MgYb_2Se_4$ Solid Electrolyte

Clarissa Glaser,[†] *Mohsen Sotoudeh,*[‡] *Manuel Dillenz,*[‡] *Kanchan Sarkar,*[‡] *Jasmin S. Bark,*[†] *Shashwat Singh,*[§] *Zhixuan Wei,*[†] *Sylvio Indris,*[‖] *Riccarda Müller,*[⊥] *Kerstin Leopold,*[⊥] *Linda F. Nazar,*[§] *Axel Groß*[‡] *and Jürgen Janek*[†]*

[†]Institute of Physical Chemistry and Center for Materials Research (ZfM), Justus Liebig University Giessen, Heinrich-Buff-Ring 17, 35392 Giessen, Germany

[‡]Institute of Theoretical Chemistry, Ulm University, Albert-Einstein-Allee 11, 89081 Ulm, Germany

[§]Department of Chemistry and the Waterloo Institute for Nanotechnology, University of Waterloo, Waterloo, Ontario N2L 3G1, Canada

[‖]Institute for Applied Materials-Energy Storage Systems (IAM-ESS), Karlsruhe Institute of Technology (KIT), Hermann-von-Helmholtz-Platz 1, 76344 Eggenstein-Leopoldshafen, Germany

[⊥]Institute of Analytical and Bioanalytical Chemistry, Ulm University, 89081 Ulm, Germany

***Corresponding author:** Juergen.Janek@phys.chemie.uni-giessen.de

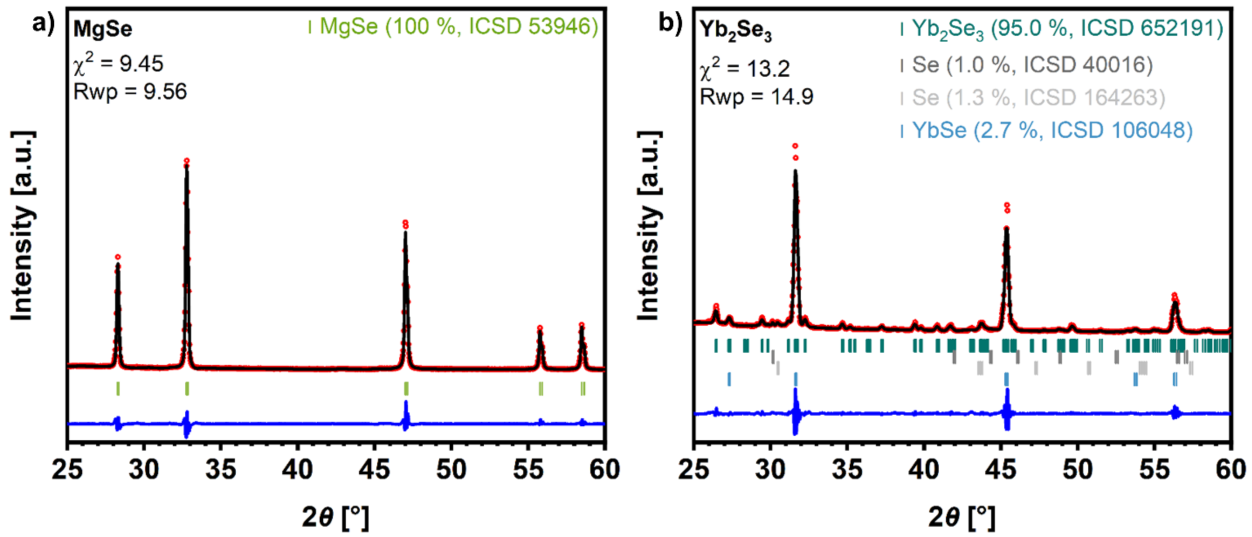

Figure S1. Rietveld refinements based on the XRD data (**Figure 1a**) of a) MgSe and b) Yb_2Se_3. Observed and calculated patterns are shown in red and black, and the difference curves are shown in blue. The refinement with respect to the MgSe sample indicates a phase-pure material without any impurities of possible phases such as Se, Mg or MgO. In the Yb_2Se_3 sample, instead, small fractions of Se (2.3 wt% in total) and YbSe (2.7 wt%) were identified, resulting from an incomplete/non-stoichiometric reaction of Yb and Se, which is expected to continue during the next reaction step ($MgYb_2Se_4$ formation).

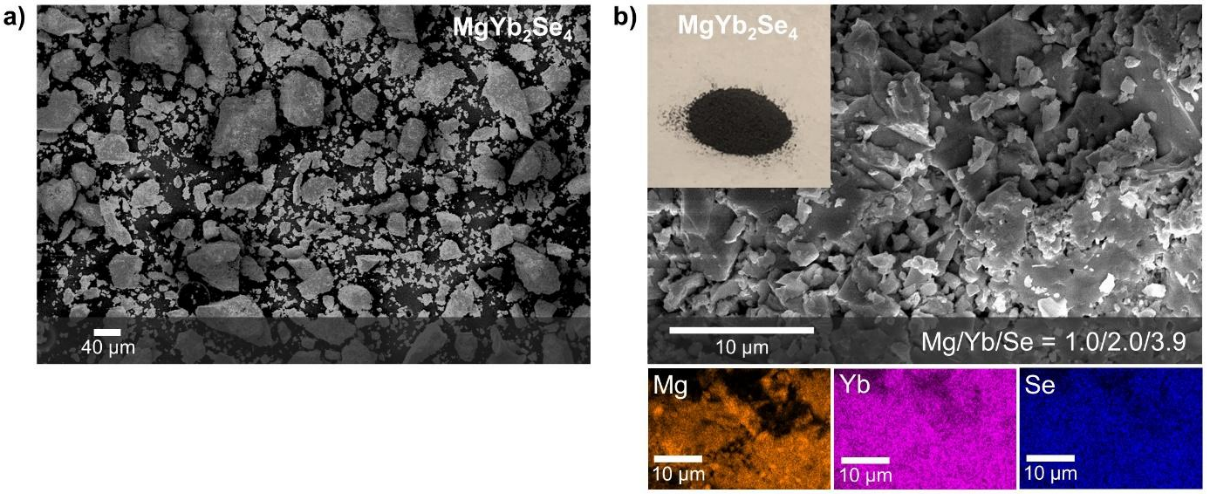

Figure S2. a) Overview SEM image showing sintered fragments of $MgYb_2Se_4$ powder with a typical size of 1–100 µm. b) Light optical image of the dark red $MgYb_2Se_4$ powder and SEM image of a $MgYb_2Se_4$ fragment (consisting of sintered 1–3 µm sized particles) with EDS mapping of the corresponding elements Mg, Yb and Se.

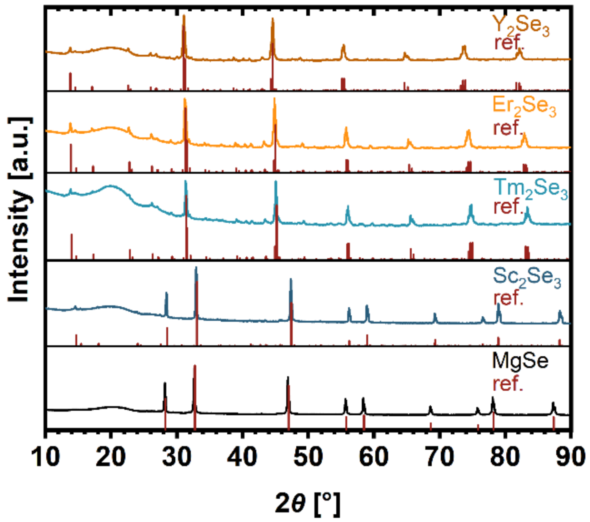

Figure S3. XRD patterns of synthesized MgSe, Sc_2Se_3, Tm_2Se_3, Er_2Se_3 and Y_2Se_3. Reproduced with permission from reference [1]. Copyright 2024, C. Glaser et al.[1]

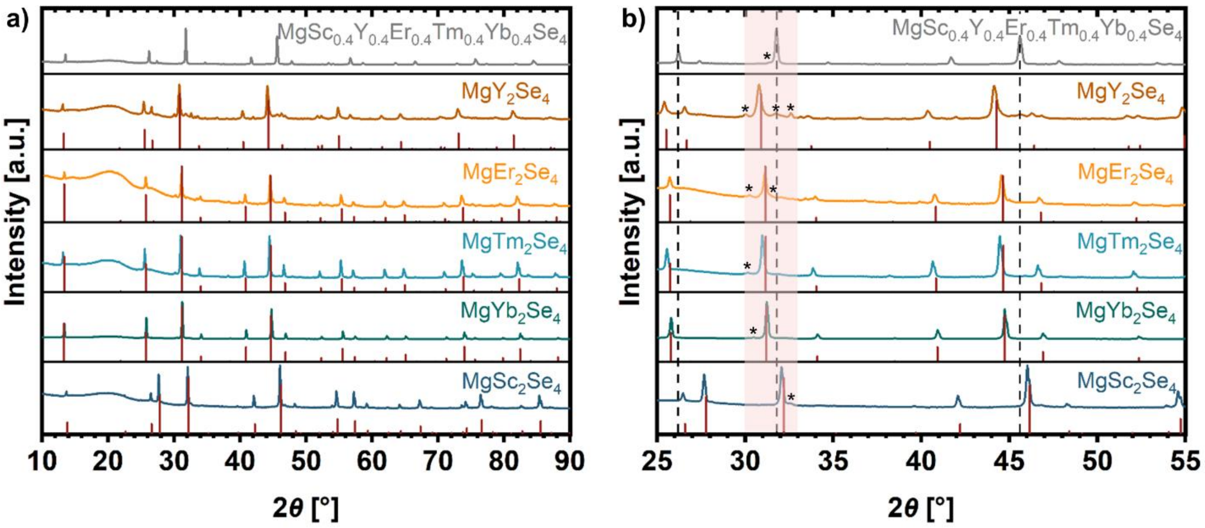

Figure S4. a) XRD patterns of spinels $MgSc_2Se_4$, $MgYb_2Se_4$, $MgTm_2Se_4$, $MgEr_2Se_4$, MgY_2Se_4 and $MgSc_{0.4}Y_{0.4}Er_{0.4}Tm_{0.4}Yb_{0.4}Se_4$ synthesized from corresponding binary selenides. All data for $MgSc_2Se_4$, $MgTm_2Se_4$, $MgEr_2Se_4$ and MgY_2Se_4 reused with permission from references [1,2]. Copyright 2023 and 2024, C. Glaser et al.[1,2] b) Zoomed view of a) showing reflections of impurities in the range of 30° to 33° marked with an asterisk. Among the spinels, $MgYb_2Se_4$ and $MgSc_{0.4}Y_{0.4}Er_{0.4}Tm_{0.4}Yb_{0.4}Se_4$ have the lowest intensities of impurity phases. The XRD pattern of $MgSc_{0.4}Y_{0.4}Er_{0.4}Tm_{0.4}Yb_{0.4}Se_4$ is quite similar to that of MgY_2Se_4 but shifted to larger angles 2θ, which is probably due to the partial substitution of the Y-position by the smaller Sc-, Yb-, Tm- and Er-ions.

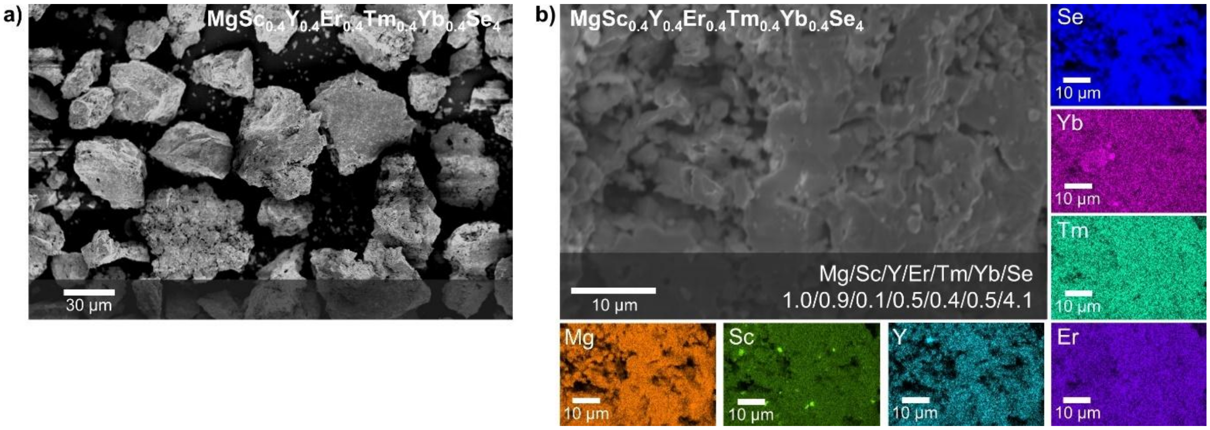

Figure S5. a) Overview SEM image of $MgSc_{0.4}Y_{0.4}Er_{0.4}Tm_{0.4}Yb_{0.4}Se_4$ powder showing sintered fragments with a typically size of 1–80 µm; and b) SEM image of a fragment with EDS mapping of the corresponding elements Mg, Sc, Y, Er, Tm, Yb and Se.

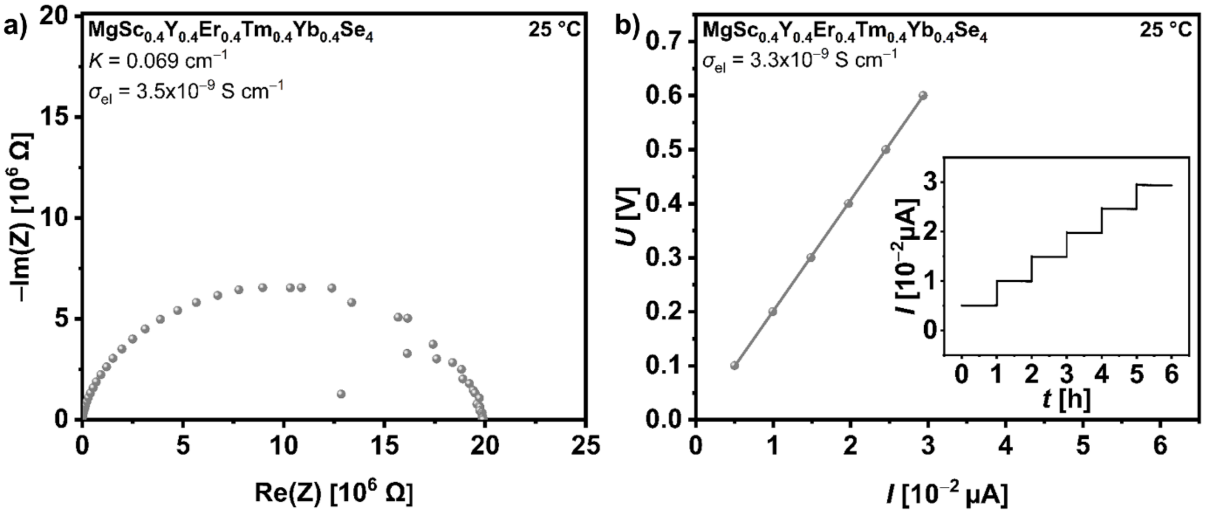

Figure S6. a) Nyquist plot of the C|$MgSc_{0.4}Y_{0.4}Er_{0.4}Tm_{0.4}Yb_{0.4}Se_4$|C press cell in the frequency range of 3 MHz to 100 mHz at 25 °C. b) DC polarization data at 25 °C obtained for the same cell configuration. During the measurement, different voltages (0.1, 0.2, 0.3, 0.4, 0.5 and 0.6 V) were held for 1 h each. The steady-state current at the end of each holding step (shown in the inset) was plotted against the corresponding voltage to calculate the electronic resistance R_{el} of the

multicationic spinel using a linear fit. As a result, $MgSc_{0.4}Y_{0.4}Er_{0.4}Tm_{0.4}Yb_{0.4}Se_4$ shows a similarly low electronic conductivity as the $MgYb_2Se_4$ spinel.

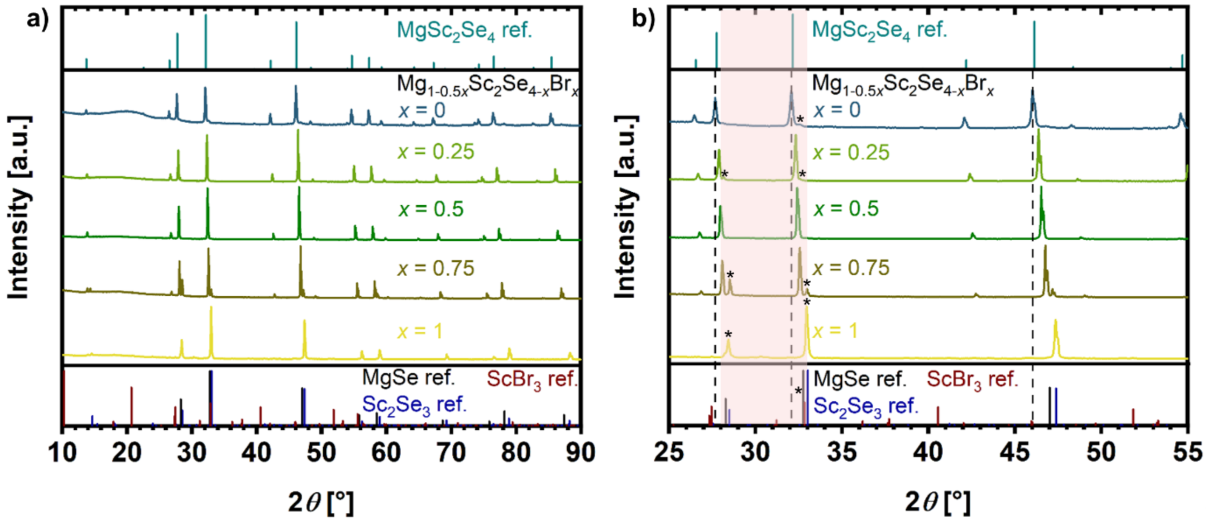

Figure S7. a) XRD patterns of $Mg_{1-0.5x}Sc_2Se_{4-x}Br_x$ compounds (x = 0, 0.25, 0.5, 0.75, 1) synthesized from binary compounds MgSe, Sc_2Se_3 and $ScBr_3$. All data for $MgSc_2Se_4$ reused with permission from reference [2]. Copyright 2023, C. Glaser et al.[2] b) Zoomed view of a) showing reflections of impurities in the range of 28° to 33° marked with an asterisk. Among the spinels, only the $Mg_{0.75}Sc_2Se_{3.5}Br_{0.5}$ spinel shows no impurity phases. The shift of the XRD patterns to larger angles 2θ with increasing x is probably due to the partial substitution of the Se-position by the slightly smaller Br-ions. At $x \geq 0.75$ decomposition to binary compounds is observed.

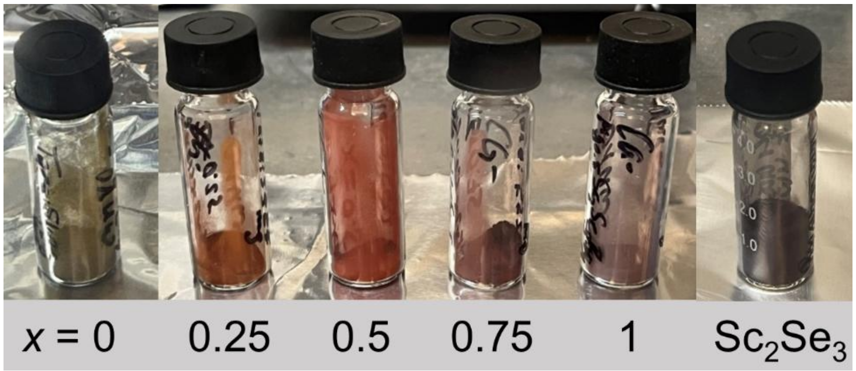

Figure S8. Light optical images of synthesized $Mg_{1-0.5x}Sc_2Se_{4-x}Br_x$ compounds (x = 0, 0.25, 0.5, 0.75, 1) and Sc_2Se_3. The color of the powder changes as x increases from gray-brown to orange, orange-red and dark red to violet. The violet color at x = 1 suggests that decomposition to the dark violet Sc_2Se_3, MgSe (white-grey) and $ScBr_3$ (white) has occurred.

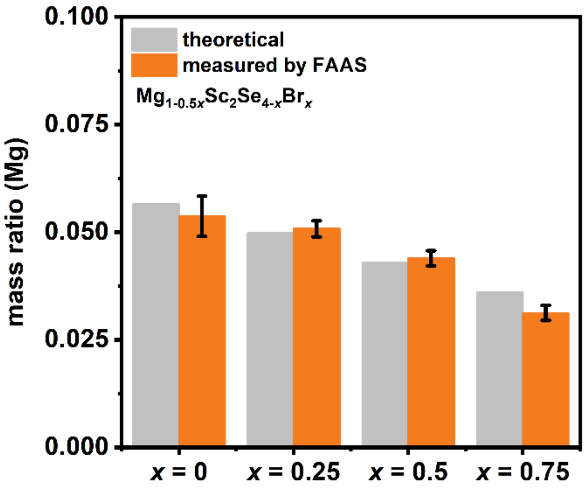

Figure S9. Theoretical mass ratio (grey) and experimentally determined mass ratio of Mg (orange) in $Mg_{1-0.5x}Sc_2Se_{4-x}Br_x$ compounds (x = 0, 0.25, 0.5, 0.75) using FAAS. Error bars represent ±1 SD with n = 3.

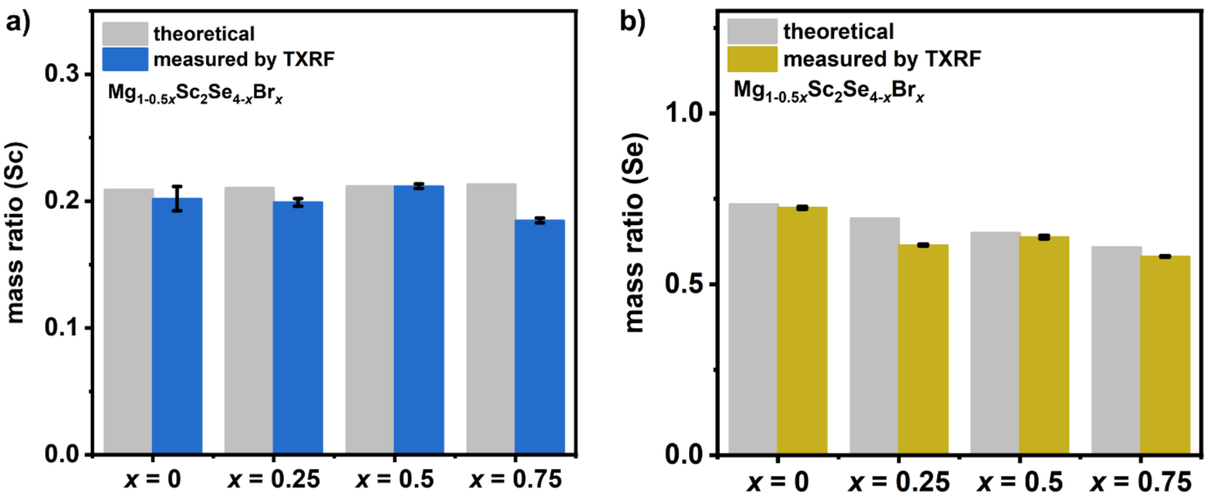

Figure S10. Theoretical mass ratio (grey) and experimentally determined mass ratio of a) Sc (blue) and b) Se (yellow) in $Mg_{1-0.5x}Sc_2Se_{4-x}Br_x$ compounds (x = 0, 0.25, 0.5, 0.75) using TXRF. Error bars represent ±1 SD with n = 3.

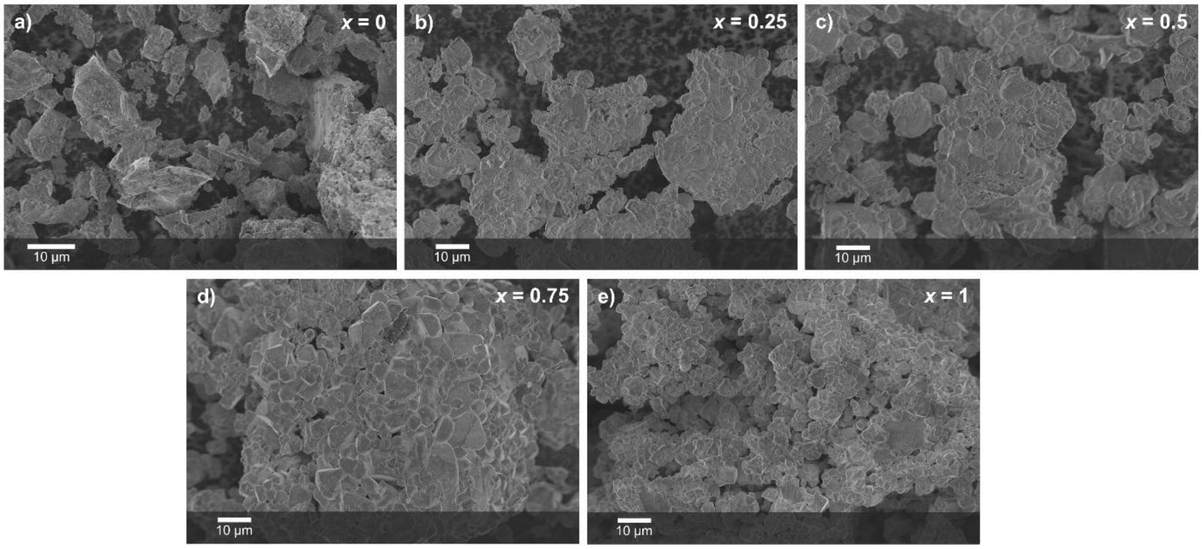

Figure S11. SEM images of $Mg_{1-0.5x}Sc_2Se_{4-x}Br_x$ (x = 0, 0.25, 0.5, 0.75, 1) showing an increase of the size of the sintered particles in the fragments from x = 0 to x = 0.75.

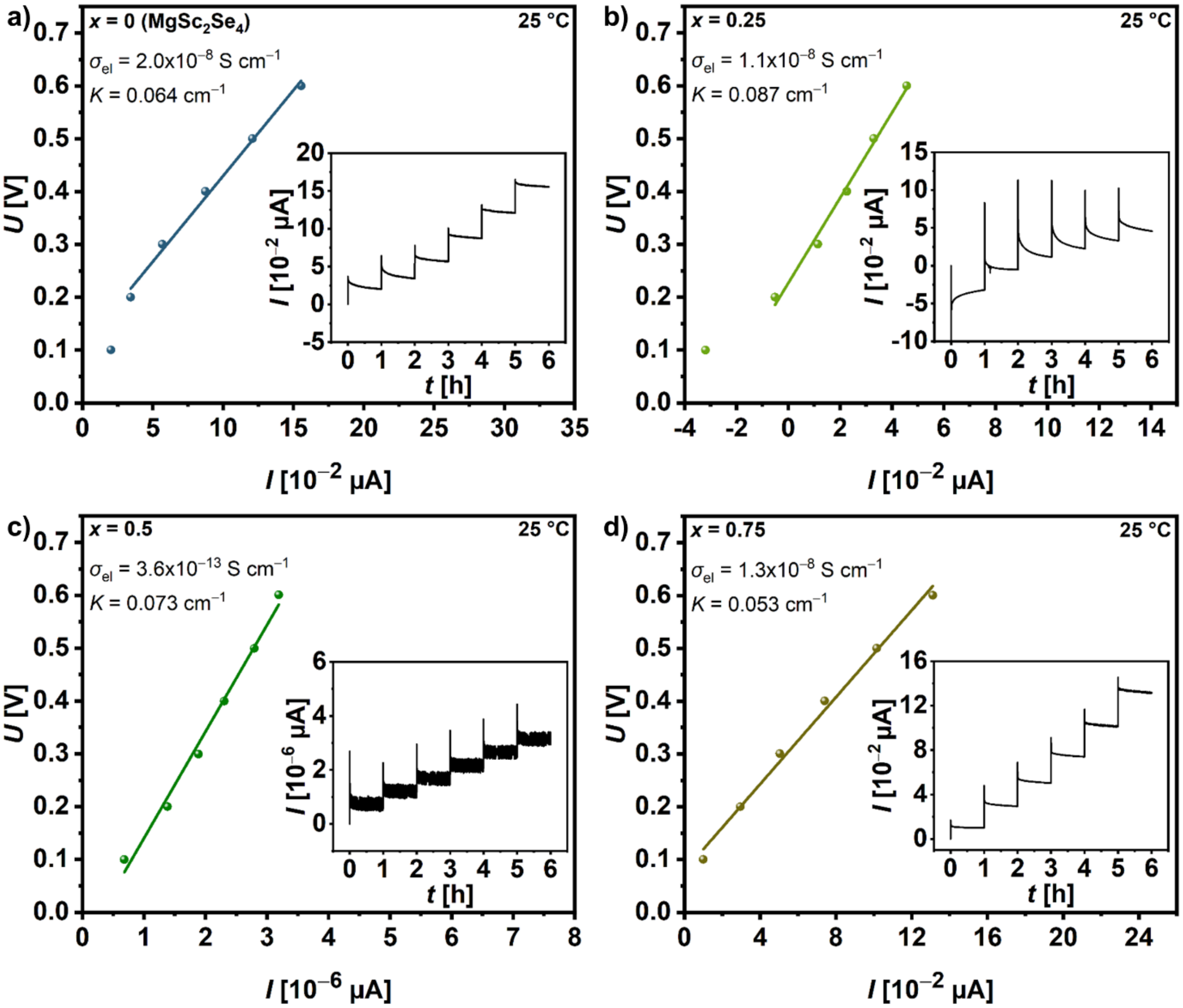

Figure S12. DC polarization data of C|$Mg_{1-0.5x}Sc_2Se_{4-x}Br_x$|C press cells obtained at 25 °C. During the measurement, different voltages (0.1, 0.2, 0.3, 0.4, 0.5 and 0.6 V) were held for 1 h each. The steady-state current at the end of each holding step (shown in the inset) was plotted against the corresponding voltage to calculate the electronic resistance R_{el} of the $Mg_{1-0.5x}Sc_2Se_{4-x}Br_x$ compounds using a linear fit. Among the spinels, $Mg_{0.75}Sc_2Se_{3.5}Br_{0.5}$ shows a five order of magnitude lower electronic conductivity.

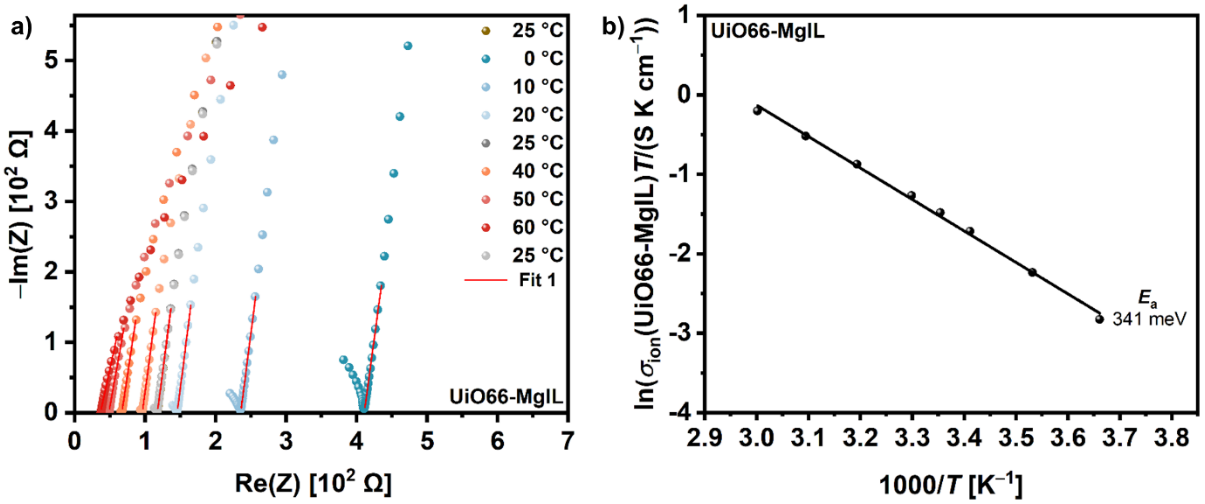

Figure S13. a) Fitted Nyquist plots of a SS|UiO66-MgIL|SS cell at different temperatures ranging from 0 °C to 60 °C and b) corresponding Arrhenius plot showing a Mg^{2+} migration barrier of 341 meV for UiO66-MgIL.

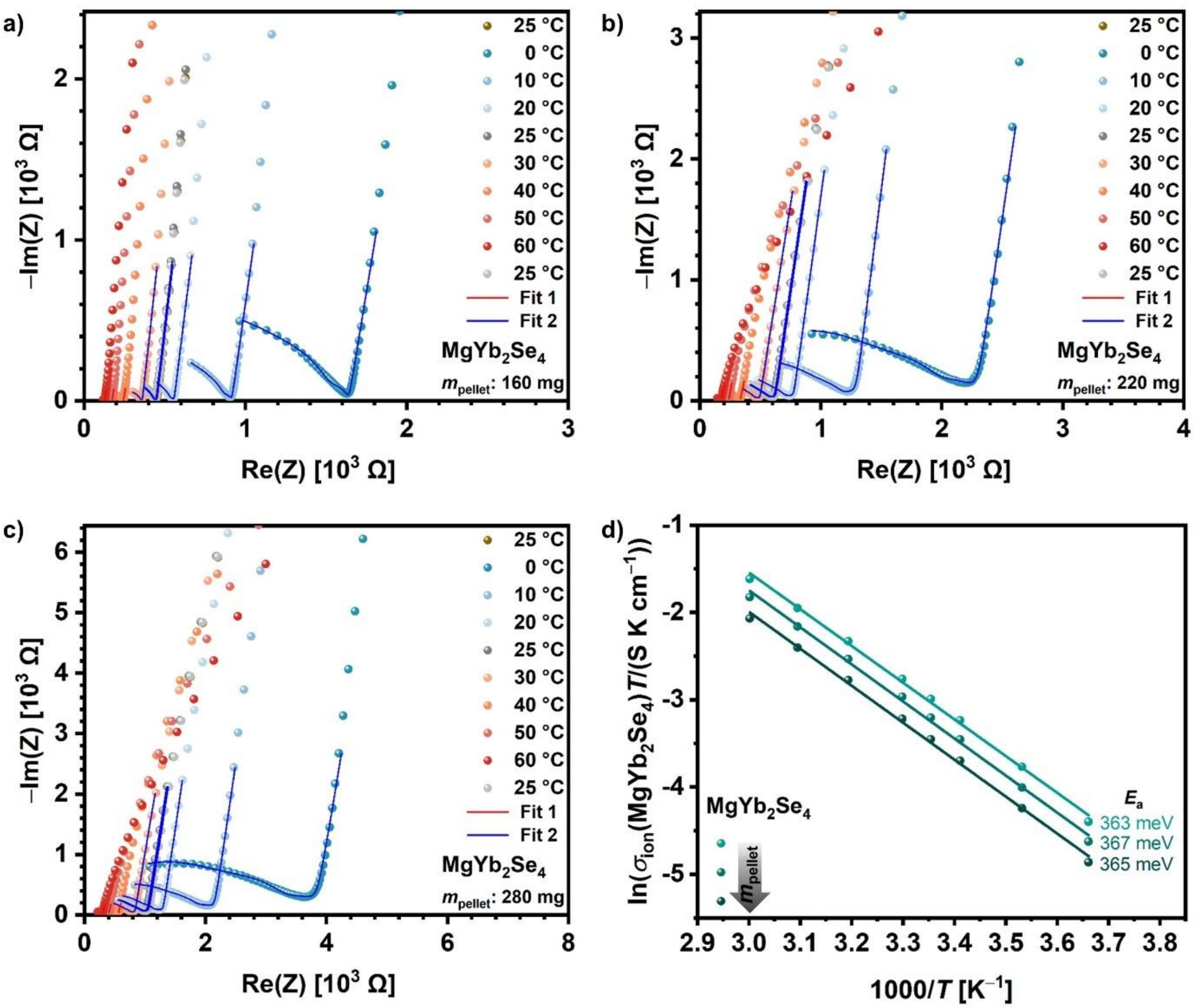

Figure S14. Fitted Nyquist plots of SS|UiO66-MgIL|$MgYb_2Se_4$|UiO66-MgIL|SS cells at different temperatures ranging from 0 °C to 60 °C using a spinel pellet mass/thickness of a) 160 mg/0.46 mm, b) 220 mg/0.54 mm, and c) 280 mg/0.76 mm; and d) Arrhenius plots of the ionic conductivity of $MgYb_2Se_4$ for each cell.

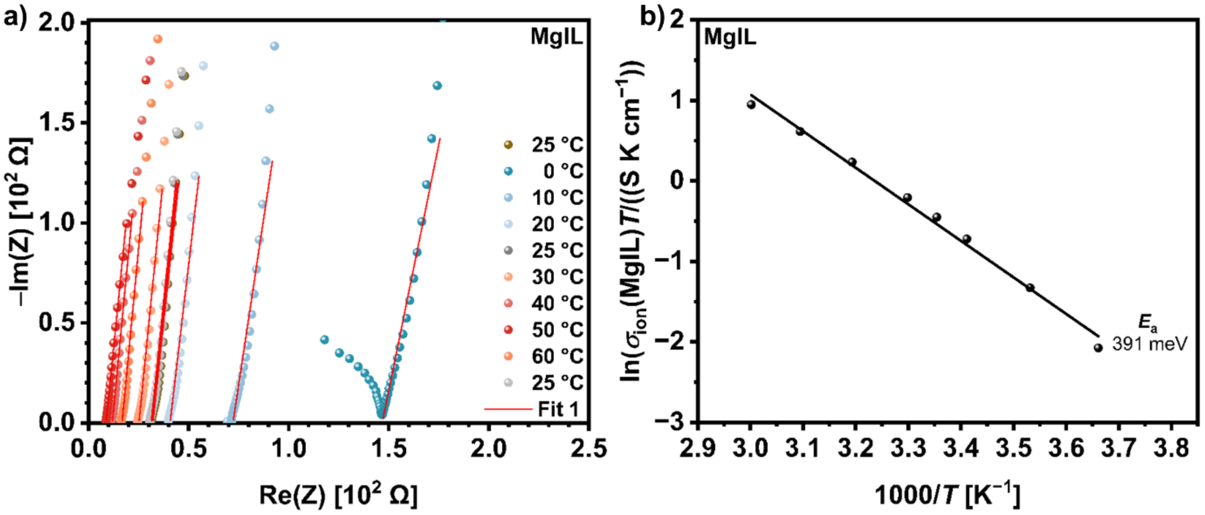

Figure S15. a) Fitted Nyquist plots of a SS|MgIL|SS cell at different temperatures ranging from 0 °C to 60 °C; and b) corresponding Arrhenius plot showing a Mg^{2+} migration barrier of 391 meV for MgIL.

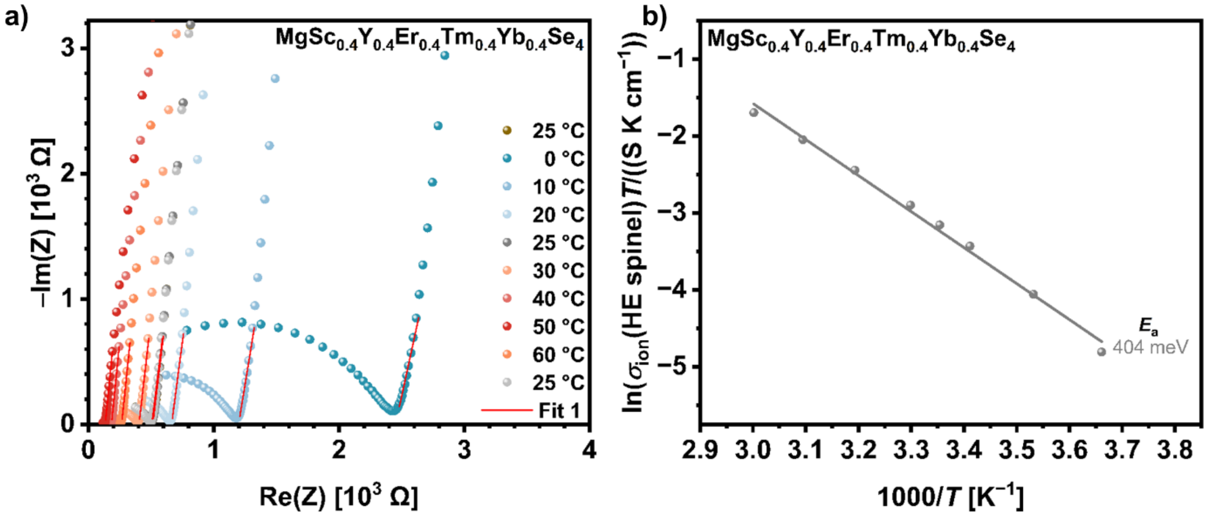

Figure S16. a) Fitted Nyquist plots of the SS|MgIL|$MgSc_{0.4}Y_{0.4}Er_{0.4}Tm_{0.4}Yb_{0.4}Se_4$|MgIL|SS cell at different temperatures ranging from 0 °C to 60 °C using a spinel pellet mass/thickness of 160 mg/0.54 mm; and d) corresponding Arrhenius plot of the ionic conductivity of $MgSc_{0.4}Y_{0.4}Er_{0.4}Tm_{0.4}Yb_{0.4}Se_4$.

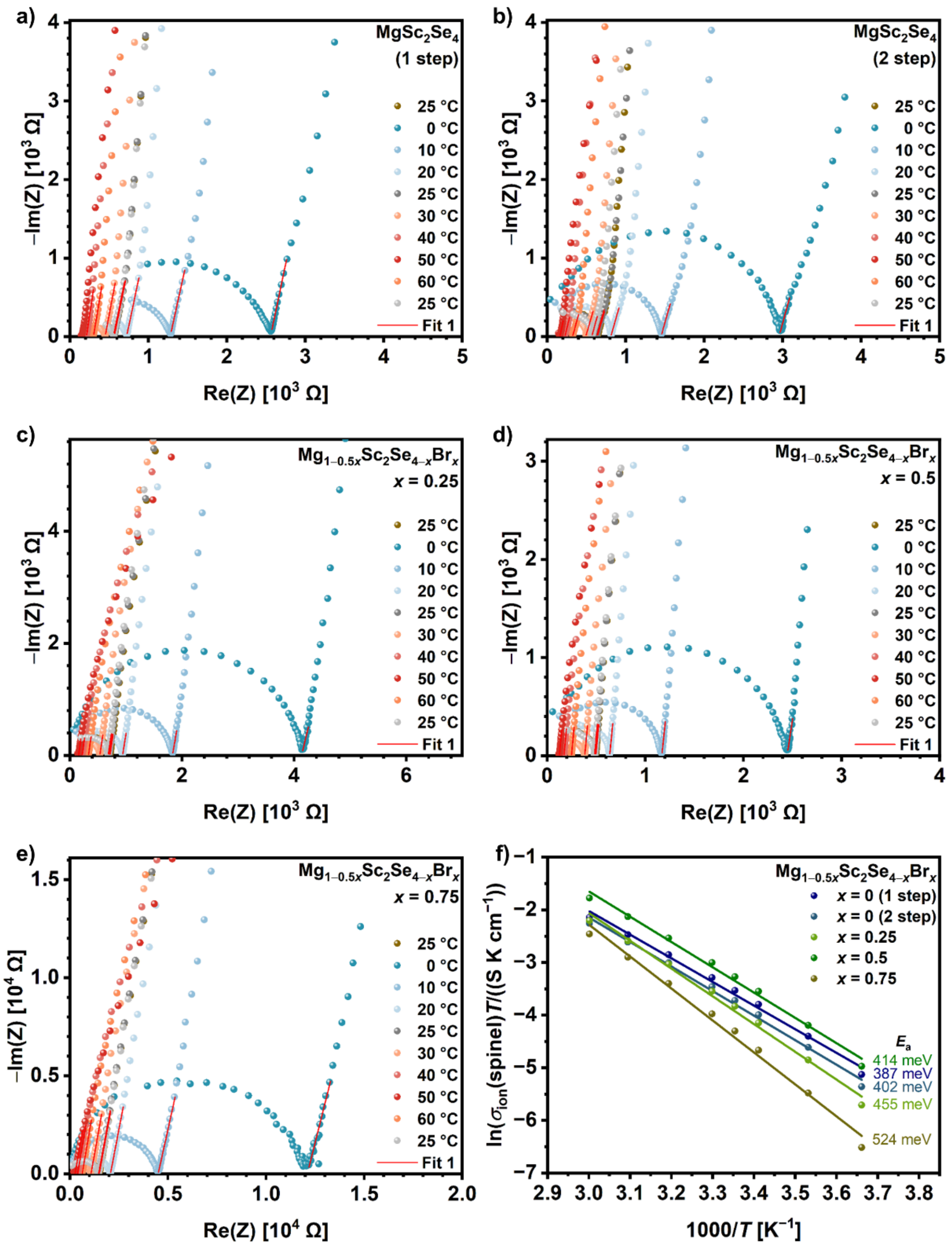

Figure S17. Fitted Nyquist plots of SS|MgIL|$Mg_{1-0.5x}Sc_2Se_{4-x}Br_x$|MgIL|SS cells at different temperatures ranging from 0 °C to 60 °C using a 160 mg spinel pellet with a thickness of a) 0.41 mm, b) 0.38 mm, c) 0.38 mm, d) 46 mm, e) 51 mm; and f) Arrhenius plots of the ionic conductivity of $Mg_{1-0.5x}Sc_2Se_{4-x}Br_x$ for each cell.

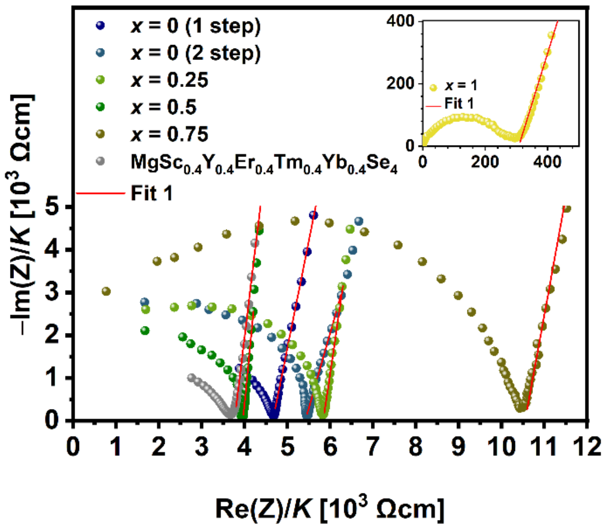

Figure S18. Overview of the fitted Nyquist plots of the SS|MgIL|$Mg_{1-0.5x}Sc_2Se_{4-x}Br_x$|MgIL|SS cells and the SS|MgIL|$MgSc_{0.4}Y_{0.4}Er_{0.4}Tm_{0.4}Yb_{0.4}Se_4$|MgIL|SS cell at 25 °C. The multicationic and multianionic ($x < 0.75$) spinels show comparable ionic resistances to the pristine $MgSc_2Se_4$ samples, while the ionic resistance increases with $x \geq 0.75$ in $Mg_{1-0.5x}Sc_2Se_{4-x}Br_x$ due to the decomposition of the spinel into binary compounds.

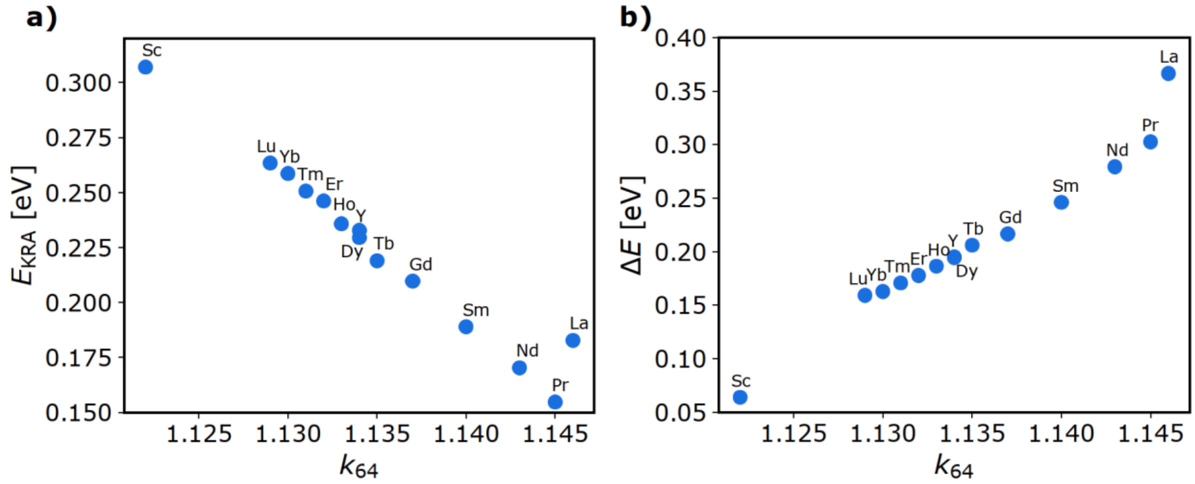

Figure S19. a) The kinetically resolved activation energy (E_{KRA}) and b) the site preference energy (ΔE), both expressed in electron volts (eV), for Mg-ion migration in the MgB_2Se_4 selenide spinel lattice as a function of the k_{64} distance ratio.

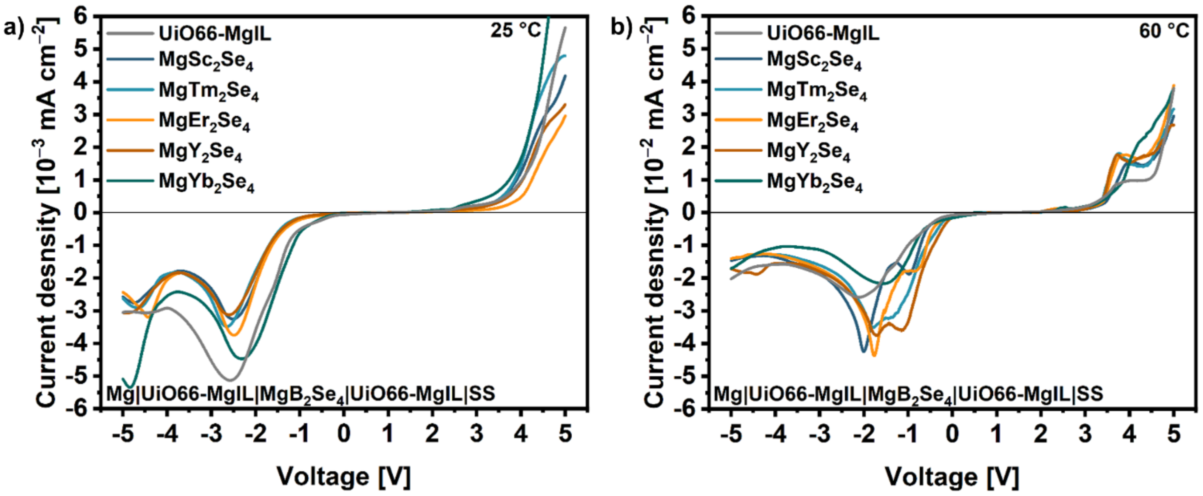

Figure S20. LSV curves of Mg|UiO66-MgIL|SS cell and Mg|UiO66-MgIL|MgB_2Se_4|UiO66-MgIL|SS cells (B = Sc, Tm, Er, Y, Yb) recorded at a scan rate of –0.1 mV s^{-1}/0.1 mV s^{-1} at a) room temperature and b) 60 °C. The current profiles of the sandwich-type cells and the reference cell without spinel layer are quite identical, as the UiO66-MgIL appears to limit the stability window. Data for $MgSc_2Se_4$, $MgTm_2Se_4$, $MgEr_2Se_4$ and MgY_2Se_4 reused with permission from reference [1]. Copyright 2024, C. Glaser et al.[1]

Table S1. Crystallographic data for $MgYb_2Se_4$ obtained from Rietveld refinement, based on the corresponding XRD pattern measured using Cu $K_α$ radiation.

Crystallographic information	Result
Crystal system	cubic
Space group	Fd-3m
Lattice parameters	$a = b = c$ = 11.45178 Å
Cell volume	1501.824 Å^3
Density	6.447 g cm^{-3}
Atomic positions of Mg	$X = Y = Z$ = 0.37500
Atomic positions of Yb	$X = Y = Z$ = 0
Atomic positions of Se	$X = Y = Z$ = 0.24456
X^2	6.27
R_{wp}	7.54
R_{exp}	3.01
Bragg R-factor	1.20
RF-factor	1.44
GoF-index	2.5

Table S2. Mass *m* and thickness *d* of $MgYb_2Se_4$ pellets and UiO66-MgIL layers (sum of both layers) in the SS|UiO66-MgIL|SS reference cell and the SS|UiO66-MgIL|$MgYb_2Se_4$|UiO66-MgIL|SS cells.

Name of cell	*m*($MgYb_2Se_4$) [mg]	*d*($MgYb_2Se_4$) [mm]	*m*(UiO66-MgIL) [mg]	*d*(UiO66-MgIL) [mm]
MOF3	0	0	80	0.70
Yb160	160	0.46	80	0.58
Yb220	220	0.54	80	0.64
Yb280	280	0.76	80	0.69

Table S3. Overview of the resistances $R1_{ion}$ (for UiO66-MgIL) and $R2_{ion}$ (for $MgYb_2Se_4$) obtained from data fitting of the SS|UiO66-MgIL|SS reference cell using the equivalent circuit in **Figure 3c** and the SS|UiO66-MgIL|$MgYb_2Se_4$|UiO66-MgIL|SS cells using the equivalent circuit in **Figure 3d**, exemplarily shown for the room temperature (25 °C) impedance measurements.

Name of cell	$R1_{ion}$ [Ω]	σ_{ion}(UiO66-MgIL) [10^{-4} S cm^{-1}]	$R2_{ion}$ [Ω]	σ_{ion}(MgB_2Se_4) [10^{-4} S cm^{-1}]
MOF3	117	7.61	-	-
Yb160	97[a]	7.61	348	1.68
Yb220	107[a]	7.61	506	1.36
Yb280	115[a]	7.61	911	1.06

For all spinel-containing cells, $R1_{ion}$ ([a]) of the UiO66-MgIL was calculated by **eq S1**. Note: The electronic resistance $R2_{el}$ of the $MgYb_2Se_4$ is not listed as it is impossible to determine reliable results by the applied equivalent circuit, described in our earlier work.[2]

Since the total UiO66-MgIL layer thickness in the SS|UiO66-MgIL|$MgYb_2Se_4$|UiO66-MgIL|SS cells can vary to those used in the SS|UiO66-MgIL|SS reference cells (see **Table S2**), the impedances of the UiO66-MgIL ($R1_{\mathrm{ion}}$ in **Table S3**) were adjusted to the layer thickness used by **eq S1**:

$$R1_{\mathrm{ion}} = \frac{d(\text{UiO66-MgIL-}i)}{d(\text{UiO66-MgIL-Ref.})} R1_{\mathrm{ion}}(\text{Ref.}) \qquad \text{(S1)}$$

i = Yb160, Yb220 and Yb280

Ref. = MOF3

Table S4. Mass *m*(spinel) and thickness *d*(spinel) of spinel pellets ($MgSc_2Se_4$, multicationic/multianionic spinels) and thickness *d*(MgIL) of the glass fiber-MgIL layers (sum of both layers) in the SS|MgIL|SS reference cell and the SS|MgIL|spinel|MgIL|SS cells.

spinel	***m*(spinel) [mg]**	***d*(spinel) [mm]**	***d*(MgIL) [mm]**
-	0	0	0.53
$MgSc_2Se_4$ (1 step)	160	0.41	0.54
$MgSc_2Se_4$ (2 step)	160	0.38	0.53
$Mg_{0.875}Sc_2Se_{3.75}Br_{0.25}$	160	0.38	0.56
$Mg_{0.75}Sc_2Se_{3.5}Br_{0.5}$	160	0.46	0.51
$Mg_{0.625}Sc_2Se_{3.25}Br_{0.75}$	160	0.51	0.58
$Mg_{0.5}Sc_2Se_3Br_1$	160	0.45	0.66
$MgSc_{0.4}Y_{0.4}Er_{0.4}Tm_{0.4}Yb_{0.4}Se_4$	160	0.54	0.53

Table S5. Overview of resistances $R1_{ion}$ (here: total ionic resistance) obtained from data fitting of the SS|MgIL|SS reference cell and the SS|MgIL|spinel|MgIL|SS cells using the equivalent circuit in **Figure 3c**, and the calculated resistances R_{ion}(MgIL) and R_{ion}(spinel), exemplarily shown for the room temperature (25 °C) impedance measurements.

spinel	$R1_{ion}$ [Ω]	R_{ion}(MgIL) [Ω]	σ_{ion}(MgIL) [10^{-4} S cm^{-1}]	R_{ion}(spinel) [Ω]	σ_{ion}(spinel) [10^{-4} S cm^{-1}]
-	32	32	21	-	-
$MgSc_2Se_4$ (1 step)	563	32[a]	21	531	0.98
$MgSc_2Se_4$ (2 step)	628	32[a]	21	596	0.81
$Mg_{0.875}Sc_2Se_{3.75}Br_{0.25}$	698	33[a]	21	665	0.73
$Mg_{0.75}Sc_2Se_{3.5}Br_{0.5}$	489	30[a]	21	459	1.3
$Mg_{0.625}Sc_2Se_{3.25}Br_{0.75}$	1464	35[a]	21	1429	0.45
$Mg_{0.5}Sc_2Se_3Br_1$	43458	39[a]	21	43419	0.013
$MgSc_{0.4}Y_{0.4}Er_{0.4}Tm_{0.4}Yb_{0.4}Se_4$	501	31[a]	21	470	1.5

For all spinel-containing cells, R_{ion}(MgIL) of the glass fiber-MgIL interlayer ([a]) was calculated from $R1_{ion}$ of the SS|MgIL|SS cell analogously to that shown for the UiO66-MgIL interlayer by **eq S1**. R_{ion}(spinel) is determined by the difference of $R1_{ion}$ and R_{ion}(MgIL). Note that σ_{ion}(spinel) may be overestimated as described in context with **Figure 4**.

REFERENCES

(1) Glaser, C.; Dillenz, M.; Sarkar, K.; Sotoudeh, M.; Wei, Z.; Indris, S.; Maile, R.; Rohnke, M.; Müller ‐ Buschbaum, K.; Groß, A.; Janek, J. MgB2Se4 Spinels (B = Sc, Y, Er, Tm) as Potential Mg ‐ Ion Solid Electrolytes ‐ Partial Ionic Conductivity and the Ion Migration Barrier. *Adv. Energy Mater.* **2024**, No. 2402269.

(2) Glaser, C.; Wei, Z.; Indris, S.; Klement, P.; Chatterjee, S.; Ehrenberg, H.; Zhao ‐ Karger, Z.; Rohnke, M.; Janek, J. To Be or Not to Be ‐ Is MgSc2Se4 a Mg ‐ Ion Solid Electrolyte? *Adv. Energy Mater.* **2023**, *13*, No. 2301980.
